# Mitochondrial Physiology in the Major Arbovirus Vector *Aedes aegypti*: Substrate Preferences and Sexual Differences Define Respiratory Capacity and Superoxide Production

**DOI:** 10.1371/journal.pone.0120600

**Published:** 2015-03-24

**Authors:** Juliana B. R. Correa Soares, Alessandro Gaviraghi, Marcus F. Oliveira

**Affiliations:** 1 Laboratório de Bioquímica de Resposta ao Estresse, Instituto de Bioquímica Médica Leopoldo de Meis, Universidade Federal do Rio de Janeiro, Cidade Universitária, Rio de Janeiro, Brazil; 2 Laboratório de Inflamação e Metabolismo, Instituto Nacional de Ciência e Tecnologia de Biologia Estrutural e Bioimagem (INBEB), Universidade Federal do Rio de Janeiro, Rio de Janeiro, RJ, Brazil; Boston University School of Medicine, UNITED STATES

## Abstract

Adult females of *Aedes aegypti* are facultative blood sucking insects and vectors of Dengue and yellow fever viruses. Insect dispersal plays a central role in disease transmission and the extremely high energy demand posed by flight is accomplished by a very efficient oxidative phosphorylation process, which take place within flight muscle mitochondria. These organelles play a central role in energy metabolism, interconnecting nutrient oxidation to ATP synthesis, but also represent an important site of cellular superoxide production. Given the importance of mitochondria to cell physiology, and the potential contributions of this organelle for *A*. *aegypti* biology and vectorial capacity, here, we conducted a systematic assessment of mitochondrial physiology in flight muscle of young adult *A*. *aegypti* fed exclusively with sugar. This was carried out by determining the activities of mitochondrial enzymes, the substrate preferences to sustain respiration, the mitochondrial bioenergetic efficiency and capacity, in both mitochondria-enriched preparations and mechanically permeabilized flight muscle in both sexes. We also determined the substrates preferences to promote mitochondrial superoxide generation and the main sites where it is produced within this organelle. We observed that respiration in *A*. *aegypti* mitochondria was essentially driven by complex I and glycerol 3 phosphate dehydrogenase substrates, which promoted distinct mitochondrial bioenergetic capacities, but with preserved efficiencies. Respiration mediated by proline oxidation in female mitochondria was strikingly higher than in males. Mitochondrial superoxide production was essentially mediated through proline and glycerol 3 phosphate oxidation, which took place at sites other than complex I. Finally, differences in mitochondrial superoxide production among sexes were only observed in male oxidizing glycerol 3 phosphate, exhibiting higher rates than in female. Together, these data represent a significant step towards the understanding of fundamental mitochondrial processes in *A*. *aegypti*, with potential implications for its physiology and vectorial capacity.

## Introduction

*Aedes aegypti* female mosquitoes are facultative blood sucking insects and this unusual dietary source is absolutely essential not only to trigger the oogenesis process, but also to transmit pathogens, such as the viruses that cause Dengue and yellow fever. Collectively, these diseases afflict nearly 400 million individuals each year globally and increasing concern on public health has prompted policies directing vectors control in endemic areas [1]. The adult individuals of *A*. *aegypti* exhibit remarkable sexual dimorphism, where males can clearly be distinguished from the females by a number of morphological and behavioral features [2]. These sexual differences can also be extended to other aspects of *A*. *aegypti* biology, such as gene expression [3, 4], hemocyte counts, and pathogen-induced immune response [5].

Dispersal of adult *A*. *aegypti* individuals plays a central ecological and medical role and field studies pointed out that a wide range of distances are covered by this insect [6,7]. Insect flight is accomplished by the contractile activity of flight muscle, which has an extremely high energy demand, and represents one of the most metabolic active tissues found in nature. The metabolic provision to power flight is diverse and the substrates required for this purpose depend on the species, flight duration, diet and aging [8–16]. For example, in honeybees carbohydrates seems to be the sole energy source to sustain flight activity [15], whereas in locusts, carbohydrates are oxidized only at the initial periods of flight, being further replaced by lipids [16]. Beyond these substrates, proline was also reported to play a key energetic role during flight activity in many insect species [11, 13, 17–24]. Interestingly, the high rates of proline oxidation observed in blood sucking insects would represent an important adaptation to blood feeding habit [25]. Also, hemolymph levels of proline are quite high in many blood feeding insect vectors of neglected tropical diseases (NTD), representing the most abundant aminoacid found in that fluid [13, 25–28]. Another important energetic substrate in flight muscle is glycerol 3 phosphate (G3P), which connects glycolysis and the ubiquinone reduction, channeling electrons from cytosol directly into the mitochondrial electron transport system [9, 12, 17, 29–34]. G3P oxidation is not only directly involved on the re-oxidation of cytosolic NADH produced during glycolysis in insect flight muscle [35], but also on the generation of mitochondrial hydrogen peroxide (H_2_O_2_) [29, 31, 34].

**Fig 1:**
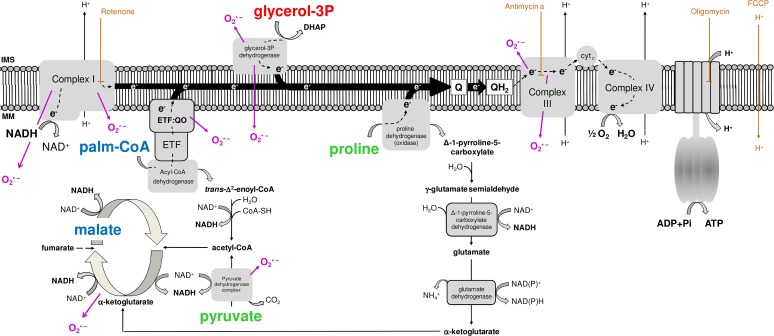
Schematic representation of the electron transport system, showing the sites of action of oxidative phosphorylation (OXPHOS) modulators (brown), the different substrates utilized throughout this study (pyruvate and proline, green; glycerol 3 phosphate, red; palmitoylcarnitine and malate, blue) and the known sites of superoxide (O2•¯) production (purple).

Since the so-called "sarcosomes" were defined as mitochondria in insect flight muscle [36], a number of studies have demonstrated the structural and functional properties of these organelles in that tissue [12, 18, 23–25, 29–37]. However, despite the dominance of insects as a major taxonomic group in number of species [38, 39], studies devoted to explore mitochondrial physiology in this important group remain largely underrepresented in the literature (only about 1.8% of all mitochondrial studies found to date in Pubmed). In this sense, a brief survey over the literature searching the respiratory activities of flight muscle mitochondria in different insect species was compiled in Table 1. We can observe that mitochondrial respiratory rates during phosphorylating conditions varies greatly among insect species and substrates, in such a way that complex I substrates contribute more importantly to oxygen consumption in some species (*Drosophila*, *Popillia*, *Manduca*), while G3P oxidation plays a prominent role in respiration in others (*Magicicada*, *Calliphora*, *Locusta* and *Bombus*). With exception of the bumblebee, all NADH-dependent respiratory rates were expressively higher in insects when compared to vertebrate heart and skeletal muscle. Similarly, G3P-mediated respiration in insect flight muscle mitochondria were in general higher than in vertebrate muscle mitochondria, with respiratory rates of *Manduca* and *Popillia* similar to vertebrate skeletal muscle, with the highest respiration observed in *Locusta* (5.6 times higher than in *Popillia*). Thus, high respiratory rates of insect flight muscle mitochondria agrees with the concept of intense oxidative metabolism in flight muscle which is essential to provide the huge energy demand posed by flight. In fact, most of the ATP required to sustain muscle contraction and flight activity are essentially provided by a highly efficient oxidative phosphorylation (OXPHOS) process within flight muscle mitochondria. These organelles play a central role in energy and redox metabolism, interconnecting the energy transduction, by means of carbohydrates, lipids and amino acids oxidation, to ATP synthesis. The electron flow at the inner membrane is channeled to ubiquinone by distinct sites, which are further transferred to complex III, cytochrome *c*, cytochrome *c* oxidase and finally to molecular oxygen by the electron transport system (Fig. 1). The energy released by electron flow is partially conserved as a proton gradient across the inner mitochondrial membrane, known as the protonmotive force (pmf), which is utilized to allow ATP generation by the F_1_F_o_ ATP synthase complex activity. Mitochondrial function is also directly involved on cellular redox balance, representing an important source of the so-called reactive oxygen species (ROS), which contributes to a number of redox-dependent signaling cascades [40]. The mechanisms involved on mitochondrial oxygen consumption and ROS generation are complex and highly regulated, and the contribution of mitochondrial physiology to basic aspects of insect biology has previously been appraised [12, 24, 25, 29, 34, 41–46]. Interestingly, recent evidence pointed out that proline oxidation promotes mitochondrial ROS formation in *Drosophila* flight muscle, which occurs essentially at levels of complex I and II [37]. Particularly relevant in the context of NTD insect vectors, recent evidence demonstrate the involvement of mitochondrial processes mediating the innate immune response against pathogens [44–46].

**Table 1:** Mitochondrial oxygen consumption of insect flight muscle compared to vertebrate muscle induced by two distinct electron transport system sites.

	NADH-dependent	G3P-dependent
**Fruit fly (*Drosophila*)** ^[^ ^12^ ^,^ ^29^ ^,^ ^31^ ^,^ ^60^ ^]^	691	327
**Cicada (*Magicicada*)** ^[^ ^30^ ^]^	290	335
**Beetle (*Popillia*)** ^[^ ^17^ ^]^	220	108
**Blowfly (*Calliphora*)** ^[^ ^61^ ^]^	200	320
**Moth (*Manduca*)** ^[^ ^63^ ^]^	265	200
**Locust (*Locusta*)** ^[^ ^32^ ^,^ ^33^ ^]^	280	611
**Bumblebee (*Bombus*)** ^[^ ^62^ ^]^	122	203
**Mosquito (*Aedes*)** ^**[**^ ^**34**^ ^]^	148	138
**Vertebrate heart** ^[^ ^64^ ^,^ ^65^ ^]^	104	-
**Vertebrate skeletal muscle** ^[^ ^65^ ^,^ ^66^ ^]^	88	162

Values of mitochondrial oxygen consumption rates during phosphorylating conditions were expressed as nmol of O_2_/min/mg protein. Data originally expressed in other units were accordingly converted to nmol of O_2_/min/mg protein, based on http://bioblast.at/images/5/5d/MiPNet12.15_RespiratoryStates.pdf. In *Drosophila*, *Locusta* and vertebrate heart and skeletal muscle, where multiple articles were utilized for calculations, an average value was obtained. NADH-dependent substrates comprise pyruvate, proline, malate and other electron donors for complex I. Superscript letters within the brackets indicate the references where data were collected.

Previous investigations of mitochondrial physiology in *A*. *aegypti* flight muscle revealed that both respiration and ADP phosphorylation were induced by tricarboxylic acid cycle intermediates, in DDT-sensitive reactions [47, 48]. Our group has shown that in females of *A*. *aegypti*, flight muscle mitochondrial oxygen consumption and hydrogen peroxide generation were transiently reduced along the blood digestion cycle [34]. Interestingly, these effects were parallel to reduced cytochrome levels and activation of mitochondrial fusion. Therefore, in an effort to understand fundamental aspects of mitochondrial physiology in this important NTD insect vector, we carried out here a systematic functional assessment of this organelle in flight muscle of young adult *A*. *aegypti* fed exclusively with sugar. This was accomplished by examining the activities of tricarboxylic acid cycle and electron transport system enzymes, the contribution of different substrates on respiration and H_2_O_2_ generation, the mitochondrial bioenergetic efficiency and capacity, the sites of H_2_O_2_ generation, as well as the differences observed in all these parameters between the sexes. The data presented here indicate that *A*. *aegypti* mitochondria utilize preferentially complex I and glycerol 3-phosphate dehydrogenase (G3PDH) substrates to sustain respiration, presenting distinct mitochondrial bioenergetic capacities among these substrates, but with preserved efficiency. In addition, the contribution of proline oxidation to respiration in female mitochondria was strikingly higher than in males. Mitochondrial superoxide (O_2_^•¯^) production is essentially mediated through proline and G3P oxidation, which takes place at distinct electron transport system points other than complex I site I_F_. Finally, sexual differences on H_2_O_2_ generation were only observed when using G3P as substrate, with male mitochondria exhibiting higher rates of O_2_^•¯^ formation than females. Therefore, the set of data described here represent a significant step towards the understanding of mitochondrial functional processes in this important insect NTD vector, with potential implications for dispersal, reproduction, survival, aging, insecticide resistance and pathogen transmission.

## Materials and Methods

### Insects

*Aedes aegypti* (Red eyes strain) were maintained at 28°C, 70–80% relative humidity with a photoperiod of 12h light/dark (L:D, 12:12h) during all life cycle. Larvae were reared on a diet consisting of commercial dog chow. Insects utilized in all experiments were adult individuals 5–7 days after the emergence. Usually about 200 insects were placed in 5 L plastic cages in a 1:1 sex-ratio and allowed to feed *ad libitum* on cotton pads soaked with 10% (w/v) sucrose solution.

### Mitochondria

Mitochondria isolation from *A*. *aegypti* flight muscle was carried out by using a method previously established by our group with minor modifications [34]. About 120 mosquitoes were immobilized by chilling on ice, dissected to obtain the thoraxes, and then gently homogenized in a 15 mL Potter-Elvehjem tissue grinder in a Teflon pestle with 10 mL of ice-cold isolation medium (250 mM sucrose, 5 mM Tris-HCl, 2 mM EGTA, 1% (w/v) fatty acid free bovine serum albumin, pH 7.4). The preparation was maintained at 4°C throughout the subsequent washing and centrifugation procedures. The liquid was centrifuged at 300 x *g* for 5 min in an Eppendorf centrifuge model 5810-R with a rotor F34-6-38. The supernatant was collected and further centrifuged at 10,000 x *g* for 10 min. The brown pellet was carefully re-suspended in approximately 0.1 mL of "respiration buffer" (120 mM KCl, 5 mM KH_2_PO_4_, 3 mM Hepes, 1 mM EGTA, 1.5 mM MgCl_2_, and 0.2% fatty acid free bovine serum albumin, pH 7.2) to give a preparation with about 20 mg of protein/mL. Protein concentration was determined by the Lowry method, using bovine serum albumin as standard [49]. Usually this method yields about 16 mg mitochondrial protein/mL/120 thoraces.

### Enzymatic activities

Enzyme activities were determined in mitochondrial preparations following methods described in the literature [50] with modifications by incubating 1.7 mg of mitochondrial preparation in 0.1 mL of hypotonic buffer (25 mM potassium phosphate and 5 mM MgCl_2_, pH 7.2) and subsequently subjected to three freeze-thawing cycles. The enzyme activities were measured at room temperature, in 1 mL of 100 mM potassium phosphate, pH 7.4 (complex I-III) or hypotonic buffer (complex IV), using a Shimadzu spectrophotometer model visible 2450 (Shimadzu Scientific Instruments, Tokyo, Japan). All enzyme activities in this study were determined using samples corresponding to about 80 μg of mitochondrial protein and at least three separate cohorts of insects. NADH:Cyt_c_ oxido-reductase activity was measured as the increase in absorbance at 550 nm due to the ferricytochrome *c* reduction. The reaction was initiated by the addition of 50 μM cytochrome *c*, 200 μM NADH, 1 mM KCN and followed by a sample of freeze-thawed mitochondria and the absorbance was monitored at 550 nm for about 10 minutes. Rotenone (0.5 μM) was added to inhibit complex I activity, which was considered as the rotenone-sensitive rate of cytochrome *c* reduction (ε = 18.7 mM^−1^ · cm^−1^). Proline:Cyt_c_ oxido-reductase was measured in the same conditions described for NADH:Cyt_c_ oxido-reductase activity, but using 25 mM of proline as substrate and 2.5 μg/mL antimycin A to inhibit complex III, instead of rotenone. G3P:Cyt_c_ oxido-reductase was measured in the same conditions described for NADH:Cyt_c_ oxido-reductase activity, but using 20 mM of *sn*-glycerol 3 phosphate as substrate and 2.5 μg/mL antimycin A to inhibit complex III, instead of rotenone. Palmitoylcarnitine (PC):Cyt_c_ oxido-reductase was measured in the same conditions described for NADH:Cyt_c_ oxido-reductase activity, but using 10 μM of palmitoylcarnitine as substrate and 2.5 μg/mL antimycin A to inhibit complex III, instead of rotenone. Cytochrome *c* oxidase activity was measured by following the decrease in absorbance due to the oxidation of ferrocytochrome *c* (ε = 18.7 mM^−1^ · cm^−1^). The reaction was initiated by the addition of freeze-thawed mitochondria and the reduction in absorbance was monitored at 550 nm. KCN (1 mM) was added to inhibit cytochrome *c* oxidase activity, which was considered as the cyanide-sensitive rate of cytochrome c oxidation. Citrate synthase (CS) activity was determined spectrophotometrically [51] by incubating a sample corresponding to 17 μg of protein from freeze-thawed mitochondria, in 75 mM Tris-HCl pH 8.0, 0.03 mM acetyl-CoA and 0.25 mM DTNB. The rate of reduced coenzyme A (CoASH) production was determined using the thiol reagent 5,5′-dithiobis (2-nitrobenzoic acid) (DTNB), which has an absorption maximum at 412 nm. The reaction was started by the addition of 0.5 mM oxaloacetate.

### Respirometry analyses on isolated mitochondria

The respiratory activity of *A*. *aegypti* flight muscle mitochondria was analyzed in a two-channel titration injection respirometer (Oxygraph-2k, Oroboros Instruments, Innsbruck, Austria) at 27.5°C. Aliquots corresponding about 200 μg of protein from freshly isolated mitochondria were transferred to the respirometer chambers containing the "respiration buffer" in a final volume of 2.2 mL and allowed to equilibrate for about 15 minutes, with continuous stirring set up at 750 rpm. Then, the oxygen concentrations and the rates of oxygen consumption were simultaneously recorded in real time in both respirometer chambers by using the DatLab 4.0 software (Oroboros Inc., Austria). The routine of electron transport system activities in *A*. *aegypti* mitochondria was carried out by following the high resolution respirometry (HRR) analyses coupled to substrate-uncoupler-inhibitor titration (SUIT) protocols established in the literature [52]. HRR-SUIT analyses were conducted using three distinct oxidizable substrate combinations as following: pyruvate + proline (Pyr+pro), *sn*-glycerol 3-phosphate (G3P), and palmitoylcarnitine + malate (PC+Mal) as indicated in different colors fonts in Fig. 1. The routine was started by the addition of substrates to final concentrations of 10 mM Pyr + 10 mM pro, 20 mM G3P, or 10 μM PC + 10 mM Mal. When using G3P, 0.5 μM rotenone was added before substrates addition in order to avoid electron backflow from glycerol 3 phosphate dehydrogenase (G3PDH) to complex I. ATP synthesis coupled to oxygen consumption through the oxidative phosphorylation (OXPHOS) was promoted by the addition of 1 mM ADP followed by a second shot reaching a final ADP concentration of 2 mM. The substrates concentration utilized in this work to induce ADP-stimulated respiration were established based on previous experiments showing that, in these conditions, oxygen consumption was maximum (data not shown). Then, 10 μM cytochrome *c* were added to each respirometer chamber and the absence of a significant stimulatory effect on respiration was used as a quality control test for integrity of the outer mitochondrial membrane [53]. The maximum non-coupled respiration was induced by stepwise titration of carbonyl cyanide p-(trifluoromethoxy) phenylhydrazone (FCCP) from 0.5 to 3 μM. The contribution of complexes I and III on electron flow were determined by the addition of 0.5 μM rotenone and 2.5 μg/mL antimycin A, respectively. All the routine experiments were carried out in a range of oxygen concentration from 240 nmol/mL to about 100 nmol/mL. Typical traces of oxygen consumption by isolated mitochondria are shown in S1 Fig. The contribution of four different dehydrogenases of the electron transport system to respiration was assessed in isolated mitochondria, by calculating the inhibitory effect of complex I (rotenone) and complex III (antimycin a) using different substrates, as following: complex I-mediated respiration was calculated as the FCCP-uncoupled pyr+pro-induced oxygen consumption rates, subtracted by the rotenone-insensitive rates. Respiration linked to proline dehydrogenase (ProDH) was determined as the rotenone-insensitive rates using pyr+pro as substrates, subtracted by the antimycin-resistant respiration. G3PDH-dependent respiration was calculated as the G3P-induced oxygen consumption rates with FCCP, subtracted by the antimycin-insensitive rates. Electron transfer flavoprotein:quinone oxidoreductase (ETF:QOR)-linked respiration was calculated as the rotenone-insensitive rates using PC+Mal as substrates, subtracted by the antimycin-resistant respiration, whereas the complex I-dependent respiration in this substrate combination was calculated as the PC+Mal-induced oxygen consumption rates with FCCP, subtracted by rotenone-insensitive rates.

### Respirometry analyses on permeabilized flight muscle

The general concept of permeabilized muscle fiber was applied to determine respiratory capacities in permeabilized flight muscle from *A*. *aegypti* [52]. Briefly, adult mosquitoes (5–7 days old), were immobilized by chilling on ice onto a pre-cooled Petri dish and then dissected to obtain the thoraces. To determine the respiratory capacities, the flight muscle were accessible by cracking the thoraces using fine forceps and then a single thorax was immediately transferred into the O2k chamber containing 2.2 mL of the "respiration buffer" (120 mM KCl, 5 mM KH_2_PO_4_, 3 mM Hepes, 1 mM EGTA, 1.5 mM MgCl_2_, and 0.2% fatty acid free bovine serum albumin, pH 7.2) supplemented with 280 U/mL of catalase. Respirometry analyses were performed at 27.5° C, using DatLab 4.0 software (Oroboros Inc., Austria), with continuous stirring at 750 rpm and all experiments started by registering the endogenous substrate supported respiration, following the HRR-SUIT protocols established in the literature [52]. In order to prevent limitations in oxygen diffusion and artificial hypoxic conditions, we carried out all experiments with whole flight muscle in an environment enriched with oxygen within the O2k chamber (O_2_ concentrations maintained between 400 and 520 nmol/mL), as recommended by the literature [52]. Experiments started by injecting a suitable amount of oxygen-enriched gaseous mixture (70% O_2_ and 30% N_2_ mol/mol) into the O2k-chamber to reach an oxygen concentration of about 500 nmol/mL. To avoid substantial drops in oxygen levels and the risk of bubble formation within the oxygraph chamber during the experiments, we injected small volumes (μL) of a H_2_O_2_ stock solution (200 mM) into the O2k-chamber. The routine of electron transport system activities in flight muscle tissue was the same utilized for isolated mitochondria and was carried out using either 10 mM Pyr+pro or 20 mM G3P as substrates. The concentrations of substrates and OXPHOS modulators were established based on the data obtained for isolated mitochondria. Typical traces of oxygen consumption by whole thorax are shown in S2 and S3 Figs. To determine the contribution of the electron transport system dehydrogenases to respiration, we utilized the same approach as described above for isolated mitochondria.

### Hydrogen peroxide (H_2_O_2_) release

Mitochondrial H_2_O_2_ production was assessed by monitoring resorufin fluorescence due to the oxidation of Amplex red (Invitrogen, USA) as described previously [34]. Briefly, 0.17 mg of mitochondrial preparations were incubated in the presence of 1.0 unit/mL horseradish peroxidase (Sigma Co., USA) on “respiration buffer” and the same substrates concentrations described for respirometry above. The rate of amplex red oxidation was recorded at room temperature using a Cary Eclipse spectrofluorimeter (Varian, USA) adapted with a continuous stirring device, operating at excitation and emission wavelengths of 530 nm and 590 nm, respectively. Typical amplex red fluorescence traces are shown in S4 Fig. Standard curves of reagent-grade H_2_O_2_ (Merck, Germany) were performed in the presence of each pharmacological OXPHOS modulator as well as of 0.17 mg of mitochondrial protein. To assess the topology of H_2_O_2_ generation sites, we utilized classical electron transport system inhibitors aiming the manipulation of the redox states of specific sites within mitochondria. Then, mitochondria were incubated with the four different substrates investigated for respirometry plus ADP, oligomycin and FCCP (5 μM) and the effects of sequential addition of rotenone and antimycin A on the amplex red fluorescence rates were registered. Specific contribution of site I_F_ of complex I to H_2_O_2_ production using pyr+pro as substrates was assessed and calculated by subtracting the rates induced by 0.5 μM rotenone from their respective rates of FCCP. Although this rotenone concentration was established by its ability to block NADH-induced cytochrome *c* reduction in submitochondrial particles of *Aedes* flight muscle mitochondria and substantiated by its capacity to inhibit respiration driven by pyr + pro or PC+Mal, it is possible that the contribution of site I_F_ of complex I to H_2_O_2_ production would be affected by higher levels of rotenone. The remaining sites of H_2_O_2_ formation promoted by pyr+pro comprised ProDH and other dehydrogenases [37, 54] that were not assigned in this work, and calculated by subtracting the rates of amplex red fluorescence induced by antimycin a by their respective rates obtained by rotenone. The contribution of G3PDH and other dehydrogenases to H_2_O_2_ generation was calculated as the amplex red fluorescence rates induced by antimycin A and then subtracted by their respective FCCP rates using G3P as substrate. The contribution of ETF:QOR and other dehydrogenases to H_2_O_2_ generation was determined by subtracting the rates of amplex red fluorescence induced by antimycin A by their respective rates obtained by rotenone, using PC+Mal as substrates. Finally, to assess the specific contribution of site I_F_ of complex I to H_2_O_2_ production using PC+Mal as substrates, the rates in the presence of rotenone were subtracted by their respective rates with FCCP.

### Data and statistics

Data in graphs were presented as bars with mean ± SD values for each condition. D´Agostino and Pearson normality tests were done for all values to assess their Gaussian distribution. Comparisons between groups were done by one-way ANOVA and *a posteriori* Tukey’s test for pair-wise comparisons. When appropriate, unpaired Student’s t-tests or Mann-Whitney´s test were employed. Differences of *p*<0.05 were considered to be significant. Correlation and linear regression analyses of data were conducted, obtaining Spearman´s or Pearson´s correlation coefficients for comparative analyses among variables. Statistical comparisons among linear regression slopes were conducted by using analysis of covariance (ANCOVA). When Gaussian distribution was achieved, outlier values were excluded by performing the Grubbs' test using the online tool available at http://graphpad.com/quickcalcs/Grubbs1.cfm. All graphs and analyses were carried out by using the GraphPad Prism software version 5.00 for Windows (GraphPad Software, USA).

## Results and Discussion

### a) Sexual size dimorphism and flight muscle mitochondrial protein recovery

Adult males and females of *A*. *aegypti* were chilly-anesthetized, their total body weight determined, as well as mitochondrial protein yields from flight muscle preparations. S1 Table shows that total body weight was significantly higher in females compared to males (*p<*0.0001) in accordance with previous studies [2]. Data of wings length and area were obtained from the literature [55, 56] and shows that both parameters were significantly higher in females (*p*<0.0001 and *p*<0.007, respectively), despite the mitochondrial protein yield were roughly the same, from about 120 individuals of each sex. The sexual size dimorphism observed here is in agreement with the general trend observed in many organisms, including most insects species [57]. These data demonstrate very similar mitochondrial protein recovery from flight muscle of both sexes.

### b) Cytochrome c is essentially reduced by means of NADH and G3P oxidation

Our first approach to investigate mitochondrial physiology in *A*. *aegypti* flight muscle was determine the activities of six distinct electron transport system enzyme complexes and citrate synthase (CS). Since several substrates are utilized as electrons sources to reduce ubiquinone, as the common intermediate electron acceptor in many organisms (see the substrates depicted in different colors on Fig. 1 and ref [58]), we assessed the activities of four different electron transport system branches coupled to cytochrome *c* reduction using NADH, G3P, proline, and PC as substrates, as well as the cytochrome *c* oxidase activity. Table 2 shows the absolute and CS-normalized electron transport system complex activities found in *A*. *aegypti* mitochondria from both sexes. We observed that cytochrome *c* oxidase activity exhibited the highest levels compared to all other electron transport system enzymes, whereas PC oxidation was unable to promote cytochrome *c* reduction. This strongly indicates that lipids are not suitable substrates to support respiration in *A*. *aegypti* flight muscle, as previously suggested [47]. CS activities were high and compatible with values obtained for other insects [59]. The electron transport system enzymatic profile normalized by CS followed a pattern very close to the absolute activity values, with G3P and NADH as the main substrates to allow cytochrome *c* reduction and cytochrome *c* oxidase exhibiting the highest activity. These data demonstrate that *A*. *aegypti* flight muscle mitochondria utilize complex I and G3P dehydrogenase (G3PDH) as the main electron fueling sites to reduce cytochrome *c*. Interestingly, all enzymatic profiles shown in Table 2 were roughly the same in both sexes, following the trend observed in mitochondrial protein recovery (S1 Table) and independent of mitochondrial mass.

**Table 2:** Enzyme activities of *A*. *aegypti* flight muscle mitochondria.

	Female	n	Male	n
**NADH:Cyt**_**c**_ **oxido-reductase**	58 ± 16	9	51 ± 23	8
**Proline:Cyt**_**c**_ **oxido-reductase**	3± 1^*b*^	3	3 ± 1 ^*d*^	3
**G3P:Cyt**_**c**_ **oxido-reductase**	79 ± 18	6	91 ± 24	6
**PC:Cyt**_**c**_ **oxido-reductase**	n.d.	3	n.d.	3
**Cyt**_**c**_ **oxidase**	164 ± 51 ^*a*^	16	189 ± 65 ^*c*^	14
**Citrate synthase**	2177 ± 1118	16	1966 ± 1148	14
**NADH:Cyt**_**c**_/**CS**	0.03 ± 0.007*	9	0.03 ± 0.01^*&*^	8
**Proline:Cyt**_**c**_/**CS**	0.001 ± 0.0006*	3	0.001 ± 0.0004^*&*^	3
**G3P-Cyt**_**c**_/**CS**	0.04 ± 0.01**	6	0.05 ± 0.01	6
**Cyt**_**c**_ **oxidase/CS**	0.07 ± 0.02	16	0.09 ± 0.03	14

Values were expressed as mean ± SD of nmol of products/min/mg protein. Statistical analyses between sexes were performed by using Student´s t or Mann-Whitney´s tests, whereas comparisons of different substrates within the same sex were carried out by using Kruskal-Wallis followed by *a posteriori* Dunn´s tests (indicated by superscript letters). Significant differences in “females” were

^*a*^ *p<0*.*01* relative to NADH:Cyt_c_ oxido-reductase,

^*b*^ *p<0*.*001* relative to Cyt_c_ oxidase. In “males” significant differences were

^*c*^ *p<0*.*01* relative to NADH:Cyt_c_ oxido-reductase and

^*d*^ *p<0*.*001* relative to Cyt_c_ oxidase. Superscript symbols represent statistical differences of all enzyme activities after normalization by citrate synthase activity as following:

* *p<0*.*001* relative to Cyt_c_ oxidase,

** *p<0*.*01* relative to Cyt_c_ oxidase,

^*&*^ *p<0*.*001* relative to Cyt_c_ oxidase. n.d. means not detected.

### c) Pyuvate+proline and G3P are the main substrates to fuel flight muscle mitochondrial oxygen consumption

Table 1 shows the compiled published data on respiration induced by complex I (NADH-dependent) and G3PDH (G3P-dependent) from flight muscle of seven different insect species, and vertebrate heart and skeletal muscle during phosphorylating conditions (ADP). A closer look on these data reveal very interesting patterns, such as the extremely high respiratory rates of *Drosophila* mitochondria when use complex I substrates [12, 29, 31, 60], as well as when *Locusta* use G3P [32, 33], pointing out the preferential substrates use to fuel flight activity. Curiously, our previous data on *A*. *aegypti* indicated that this insect utilized almost equally complex I (using pyr+pro) and G3PDH (G3P-dependent) substrates to fuel oxygen consumption [34]. Considering complex I substrates, *A*. *aegypti* flight muscle respiratory rates were markedly lower compared to most insect species, resembling the rates observed in bumblebee [62] and vertebrate mitochondria [64–66]. Thus, in order to improve our understanding of mitochondrial respiratory capacities and substrates dependences in *A*. *aegypti* flight muscle mitochondria, we utilized HRR-SUIT protocols for this purpose [52].

S1 Fig. shows representative oxygen flux traces of flight muscle mitochondria of both *A*. *aegypti* females and males during typical HRR-SUIT experiments. The routine described in the methods section was applied to assess the contribution of three different substrates combinations: 10 mM Pyr+Pro; 20 mM G3P, 10 μM PC + 5 mM Mal on oxygen fluxes in isolated mitochondria from females (Table 3) and males (S2 Table) of *A*. *aegypti*. When mitochondria from females (Table 3) were incubated only with substrates ("Leak") the respiratory rates were in general low. This metabolic state is defined by a non-phosphorylating respiratory condition that is essentially limited by the magnitude of the protonmotive force (pmf), and the respiratory rates are compensated by the proton leak, relieving the inhibitory effect of high pmf on the oxygen flux. The highest oxygen fluxes on both phosphorylating (ADP) and uncoupled (FCCP) metabolic states in female mitochondria were obtained when using Pyr+Pro as substrates, followed by G3P, and PC+Mal. Interestingly, the high respiratory rates induced by Pyr+pro may explain the complete depletion of glycogen stores in fat body and flight muscle after flight to exhaustion [67]. On the other hand, the low rates of oxygen fluxes induced by PC+Mal strongly indicates that fatty acid oxidation is not a major pathway to provide the energy required to sustain *A*. *aegypti* flight activity, which is in contrast to other insect species [63]. The limited capacity of *A*. *aegypti* flight muscle mitochondria to use fatty acid oxidation to sustain respiration shown in Tables 3 and S2 is in line with the undetectable capacity of PC to promote cytochrome *c* reduction (Table 2). It is long known that Dipteran insects, the order which belong *A*. *aegypti* and other mosquitoes, utilize mostly carbohydrate (glucose) and aminoacid (proline) as substrates to sustain flight activity, exhibiting respiratory quotients close to unity [67,68]. Indeed, glycogen stores were depleted in the fat body and flight muscle of *Culex* mosquitoes after flight to exhaustion, despite the fat deposits remained stable [67]. Later, it was demonstrated that particulate fractions of *Aedes* flight muscle were unable to oxidize -hydroxybutyrate [47]. More recently, a comprehensive study demonstrated that *Anopheles stephensi* mosquitoes were unable to use ketone bodies, as well as octanoate and octanoylcarnitine to sustain respiration [35]. Interestingly, comparisons of fatty acid oxidation of these mitochondria with those from locust and mammalian muscle revealed that octanoylcarnitine oxidation is fairly high in these latter two, but completely absent in *Anopheles* mitochondria [35]. Also, despite carnitine play a key role in allowing fatty acid oxidation in flight muscle of some insects [69], its presence and metabolism cannot be directly assumed as a proxy of fatty acid oxidation capacity. A good example in this regard is the blowfly *Phormia regina*, which is unable to oxidize fatty acids to sustain respiration [70], but exhibit high levels of carnitine as well as an active acetyl carnitine transferase [70]. Unexpectedly, carnitine in this insect revealed to be important for pyruvate metabolism by allowing acetylcarnitine formation from pyruvate decarboxylation, which prevent CoA and ATP depletion [70]. In this sense, we think that limited fatty acid oxidation in *A*. *aegypti* flight muscle mitochondria is not related to CoA depletion, since most experiments conducted here (with the exception of Table 2) were carried out in the presence of malate, which generates oxaloacetate and then allow CoA recycling by promoting CS reaction. Therefore, the low contribution of fatty acid to respiration in *A*. *aegypti* mitochondria is not related to a specific substrate, co-factors depletion or the availability/utilization of other fatty acids. Rather, our data strength the general trend observed in all Dipteran insects that fatty acid oxidation would play a minor (if any) role on respiration in flight muscle mitochondria of these particular group of insects.

**Table 3:** Contribution of different substrates to respiration of isolated mitochondria from flight muscle of *A*. *aegypti* females.

Metabolic state	Pyr+Pro	n	G3P	n	PC+Mal	n
Leak	15 ± 7	11	30 ± 11	9	2 ± 1 ^*a*^	8
+ ADP	132 ± 58 ^#^	11	68 ± 28 **	9	6 ± 2	8
+ FCCP	157 ± 64 ^#^	10	100 ± 42 * ^,^ ^@^	9	8 ± 2	8
+ Rotenone	21 ± 12	11	-		1 ± 1	8
+ Antimycin	7 ± 4	10	8 ± 4	9	1 ± 1	8

Values were expressed as mean ± SD of nmol oxygen consumed/min/mg protein with the following substrates: 10 mM pyruvate + 10 mM proline (Pyr+Pro), 20 mM *sn* glycerol-3 phosphate (G3P) or 10 μM palmitoylcarnitine + 5 mM malate (PC+Mal). Addition of OXPHOS modulators were indicated as "+" in the first column as following: 2 mM ADP (+ADP), 10 μM cytochrome c (not shown), 2 μM FCCP (+FCCP), 0.5 μM rotenone (+Rotenone), and finally 2.5 μg/mL antimycin A (+ Antimycin). For all G3P measurements, experiments started after the addition of 0.5 μM rotenone. Statistical analyses were carried out only between the groups of different substrates and mitochondrial metabolic state and were performed by using either Kruskal-Wallis test followed by *a posteriori* Dunn´s test (indicated by superscript letters) or by ANOVA and *a posteriori* Tukey´s test (indicated by superscript symbols). Significant differences in “Leak” were

^*a*^ *p<0*.*0001*, relative to Pyr+Pro and G3P. In “ADP”, significant differences were

** *p<0*.*01* relative to Pyr+Pro and PC+Mal,

^#^ *p<0*.*001* relative to PC+Mal. In “FCCP”, significant differences were

* *p<0*.*05* relative to Pyr+Pro, ^#^ *p<0*.*001* relative to PC+Mal,

^@^ *p<0*.*001* relative to PC+Mal.

**Fig 2:**
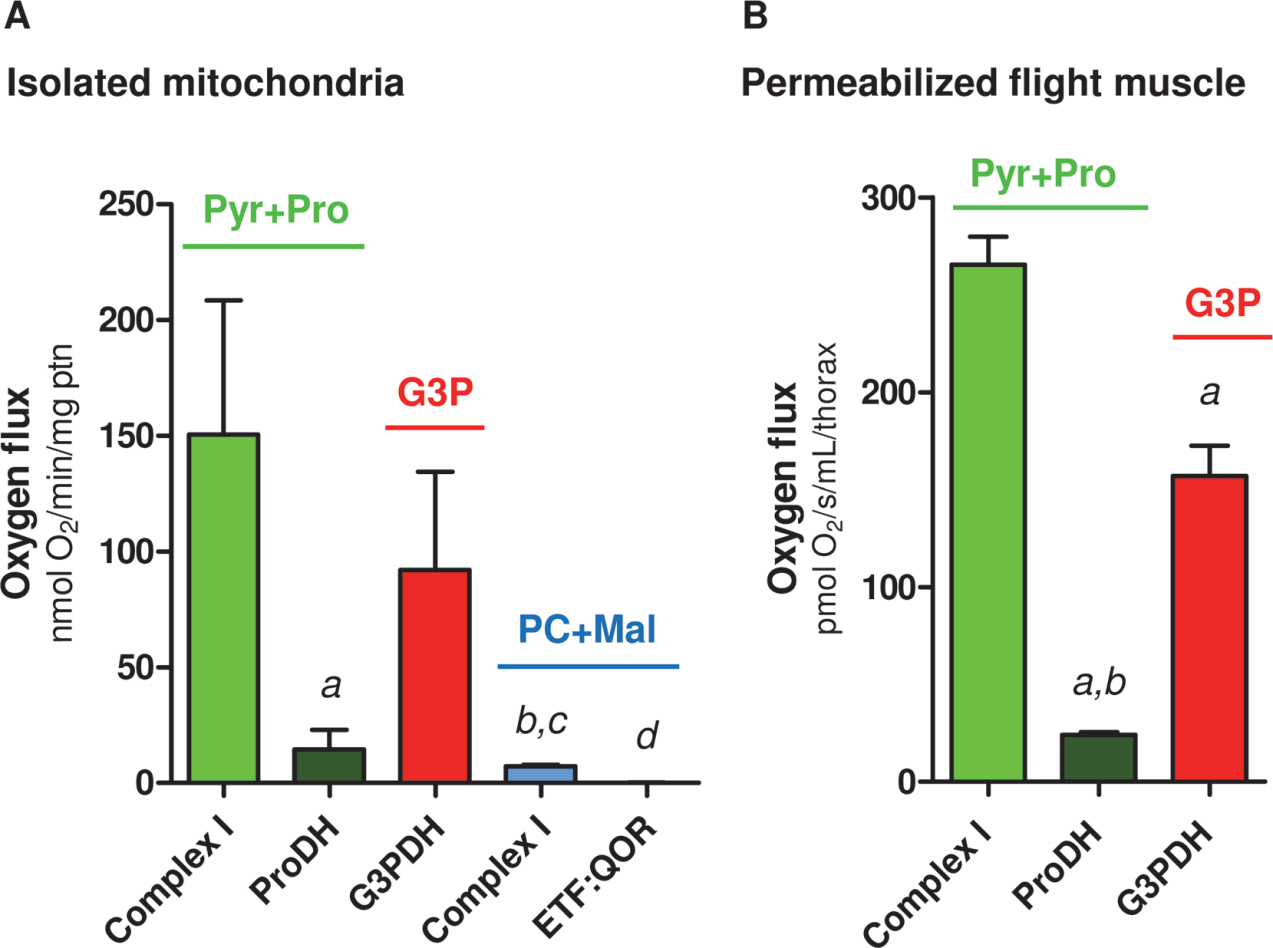
Complex I and G3PDH represent the major electron donor sites to support respiration in female *A*. *aegypti* flight muscle. Oxygen consumption rates from female *A*. *aegypti* isolated mitochondria (A) and permeabilized flight muscle (B) were calculated from values shown in Tables 3 and 4. Data are expressed as mean ± SD of at least seven different experiments. Comparisons between groups were done by Kruskal-Wallis and *a posteriori* Dunn's tests. Figure (A): ^*a*^ *p*<0.05 relative to Complex I Pyr+pro; ^*b*^ *p*<0.05 relative to G3P; ^*c*^ *p*<0.01, relative to Complex I Pyr+pro; ^*d*^ *p*<0.001, relative to Complex I Pyr+pro and G3P. Figure (B): ^*a*^ *p*<0.001 relative to complex I; ^*b*^ *p*<0.001 relative to G3P.

Proline oxidation plays a key energetic role to sustain flight activity in many insect species [37,71] including *A*. *aegypti* [13, 34]. In this sense, Figs. 2 and S5 shows that the contribution of different dehydrogenases on the maximum respiratory rates is essentially maintained by complex I, using pyr+pro as substrates, and G3PDH activities (Fig. 2A: females > 94%; S5A Fig.: males > 97% of total), whereas the direct contribution of ProDH, complex I (when using PC+Mal as substrates) and ETF:QOR together had a minor role on respiratory rates (Fig. 2A: females > 5.7%; S5A Fig.: males > 2.8% of total). Indeed, in Table 3 we demonstrate that at least 90% of electrons are directed to electron transport system through complex I activity when *A*. *aegypti* flight muscle mitochondria utilize Pyr+Pro as substrates, based on the inhibitory effect of rotenone during uncoupled state. It is important to consider that part of these electrons are also derived from mitochondrial proline metabolism since the oxidation of this aminoacid occurs at four distinct sites (Fig. 1) with the first one catalyzed by ProDH, channeling the electrons directly to ubiquinone and then to molecular oxygen by the electron transport system. The other three steps occur at Δ-1-pyrroline-5-carboxylate dehydrogenase, glutamate dehydrogenase and the tricarboxylic acid cycle enzymes, which are all coupled to NADH generation [72]. Complex I also was the major route of electrons entry at the electron transport system when using PC+Mal as substrates, but with a strikingly reduced contribution to respiration in absolute values when compared to Pyr+Pro (Fig. 2A, complex I at Pyr+pro *vs*. complex I at PC+Mal). Finally, similar trends on substrate dependence of respiratory rates were observed in flight muscle mitochondria from *A*. *aegypti* males, with complex I, using pyr+pro as substrates, and G3PDH the major sites of electron entry at the electron transport system (S5A Fig.). Remarkably, the contribution of ProDH to respiration in male mitochondria was lower than in females, suggesting that metabolism of this aminoacid is distinct among sexes. The sexual differences related to mitochondrial physiology, and the respiration driven by proline oxidation will be addressed later in this manuscript.

### d) Mitochondrial physiology in A. aegypti flight muscle can be studied in situ without organelle isolation

We next investigated the possibility to study mitochondrial physiology *in situ* using whole flight muscle from individual *A*. *aegypti* insects, instead of isolated mitochondria. Similar approaches have also been applied to study flight muscle mitochondria in *Drosophila* [73–76] and revealed the enormous potential of this methodology to investigate mitochondrial function in a more physiological way. This approach has a number of advantages, allowing: *i)* the study of mitochondrial function in the natural cellular environment; *ii)* the preservation of contacts between mitochondria and other organelles; *iii)* the assessment of mitochondrial oxygen consumption using reduced number of insects, and *iv)* the bypass of time-consuming and stressful methods employed to obtain enriched mitochondrial preparations. For this sake, we developed a procedure (see methods section) to measure *in situ* substrate-induced oxygen consumption rates on whole *A*. *aegypti* flight muscle, based on the literature [74–76]. During our first experiments, we observed that physical permeabilization of flight muscle, provided by the magnetic stirring of the respirometer, was enough to allow free access of substrates and OXPHOS inhibitors on *A*. *aegypti* flight muscle, without affecting mitochondrial structure (data not shown). In order to avoid respiratory limitation in flight muscle bundles due to restricted oxygen diffusion, we maintained the oxygen tension of about 450 nmol/mL during all measurements [52]. S2 Fig. show two representative HRR-SUIT oxygen flux traces of a single *A*. *aegypti* thorax from female (S2A Fig.) or male (S2B Fig.) using Pyr+Pro as substrates. Interestingly, the effects of all OXPHOS modulators on oxygen consumption observed on isolated mitochondria (S1 Fig.) were replicated using permeabilized flight muscle, in both sexes using Pyr+Pro (S2 Fig.) or G3P as substrates (S3 Fig.). We then investigated the respiratory pattern of permeabilized flight muscle using only these two substrates, since they induced the highest cytochrome *c* reduction and respiratory rates in isolated mitochondria (Tables 2, 3 and Figs. 2A, S2, S5A). We assessed the quality of our measurements in terms of structural intactness of mitochondria, by evaluating the effects of two compounds on oxygen fluxes: *i)* ADP, which would reflect the OXPHOS functionality, and *ii)* cytochrome *c*, which increase the respiratory rates when the structure of mitochondrial outer membrane is compromised [52]. In Table 4, we observed that when female flight muscles were incubated only with substrates ("Leak" condition) the respiratory rates induced by G3P were significantly higher when compared to Pyr+Pro (*p*<0.001), following the same pattern with isolated mitochondria (Table 3, "Leak" at G3P *vs*. Pyr+Pro). "Leak" respiratory rates were also significantly higher in male flight muscle when using G3P compared to Pyr+Pro (*p*<0.001) (S3 Table). Also, ADP significantly increased the respiratory rates, regardless the substrate and sex, and cytochrome *c* caused no significant change on oxygen fluxes, indicating the preserved structure of mitochondria in permeabilized flight muscle (S2 and S3 Figs). The respiratory rates during phosphorylating (ADP) and uncoupled (FCCP) conditions were significantly higher (*p*<0.001) when using Pyr+pro as substrates than with G3P (Table 4). Interestingly, maximum respiration (uncoupled by FCCP) induced by G3P correspond to only 43.7% of Pyr+Pro respiratory rates, strengthening the preference of these substrates to sustain the high energy demands posed by flight. We also observed that under ADP phosphorylation, the respiratory rates of permeabilized flight muscle using Pyr+Pro and G3P represented 88.3% and 88% of uncoupled oxygen consumption, respectively, indicating that ATP synthesis in *A*. *aegypti* flight muscle demand a large proportion of the maximum respiratory capacity regardless the substrate. Rotenone and antimycin-insensitive oxygen consumption rates represented 11.4% and 3.4%, respectively, of uncoupled respiration, indicating that at least 88% of the electrons in this substrate combination are channeled to the electron transport system by complex I. Indeed, Figs. 2B and S5B shows that the contribution of different dehydrogenases on the maximum respiratory rates in permeabilized muscle, which is essentially maintained by complex I, using Pyr+pro, and G3PDH activities in both sexes (Fig. 2B: females > 94%; S5B Fig.: males > 98% of total), whereas the direct contribution of ProDH to respiration plays a minor role (Fig. 2B: females > 5.3%; S5B Fig.: males > 1.4% of total). Similarly to what we found on isolated mitochondria, proline oxidation coupled to respiration was more prominent in females than in males, strengthening the concept that females were more adapted to utilize this aminoacid to sustain respiration [13, 25]. Finally, a comparison of respiratory data obtained for whole flight muscle and isolated mitochondria for both sexes using Pyr+pro or G3P (S6 Fig.) revealed the striking proportionality of oxygen fluxes obtained in both approaches, clearly indicating that mitochondrial physiology in *A*. *aegypti* flight muscle can be assessed *in situ* without organelle isolation.

**Table 4:** Contribution of different substrates to sustain respiration in permeabilized flight muscle from *A*. *aegypti* females.

Metabolic state	Pyr+Pro	n	G3P	n
Leak	42 ± 10	13	81 ± 20*^a^*	12
+ ADP	265 ± 45	13	140 ± 43 *^a^*	12
+ FCCP	300 ± 50	13	169 ± 55 *^a^*	12
+ Rotenone	34 ± 10	13	-	
+ Antimycin	10 ± 6	13	12 ± 4	12

Values were expressed as mean ± SD of pmol O_2_/s/mL/thorax in five different mitochondrial metabolic states using: 10 mM pyruvate + 10 mM proline, 20 mM *sn* glycerol-3 phosphate, followed by the addition of 2 mM ADP (ADP), 10 μM cytochrome c (not shown), 2.5 μM FCCP, 0.5 μM rotenone, 2.5 μg/mL antimycin A. Statistical analyses were performed using Mann-Whitney test.

^*a*^ *p<0*.*001* relative to Pyr+Pr*o*.

### e) Flight muscle exhibit similar mitochondrial bioenergetic efficiencies and distinct capacities among substrates

Mitochondrial ATP production is determined by their bioenergetic efficiency (defined as the ATP produced in mitochondria per molecule of nutrient) and their ATP synthesis capacity (defined as the rate of ATP produced in mitochondria per unit of time), which are both regulated by the cellular energy demand and supply. Assuming that insect flight plays a central ecological role to reproduction and dispersal, representing one of the most energy demanding processes in Animal Kingdom [77], flight muscle high respiratory capacity must be tightly coupled to ATP synthesis. In this regard, one can speculate that efficiency and capacity of mitochondrial ATP production would vary among different substrates, being regulated by *i)* the substrate transport to mitochondria; *ii)* the oxidation potential provided by the mitochondrial dehydrogenases and the electron transport system complexes and *iii)* the degree of electron transport system coupling to OXPHOS.

Comparative analyses of respiration among insects indicate that oxygen consumption in *A*. *aegypti* flight muscle is in general lower than any other insect species, regardless the substrate utilized and sex. For example, Pyr+pro (NADH-dependent)-supported oxygen consumption rates in *A*. *aegypti* flight muscle were about 12% and 84% lower than in *Bombus* and *Drosophila*, respectively (Tables 1, 3 and S2). In addition, G3P-induced respiration in *A*. *aegypti* was 77% and 58% lower than *Locusta* and *Magicicada* flight muscle mitochondrial oxygen consumption rates, respectively (Tables 1, 3 and S2). Conceivably, the dispersal potential of *A*. *aegypti* would be limited by their lower flight muscle aerobic capacity, when compared to other insect species. Supporting this idea, a recent study demonstrated that butterfly species with long-distance fly behavior had much higher aerobic capacity, presenting high cytochrome *c* oxidase activity and content, and also with larger and more numerous mitochondria than shorter-distance flyers [78]. We thus assumed that in a condition with high energy-demand, such as in active insect flight muscle, respiratory rates coupled to ATP synthesis (defined as the respiratory rate induced by ADP, or OXPHOS) would be close to the maximum respiratory rates provided by uncoupling, varying in a proportional way. Therefore, calculating the OXPHOS values and correlating them with their respective maximum uncoupled respiratory rates values would give us an insight of both mitochondrial bioenergetic capacity (OXPHOS) and efficiency (slope). Based on these assumptions, we calculated the OXPHOS respiratory rates (by subtracting the ADP oxygen fluxes by their respective leak rates), and the maximum uncoupled respiratory rates (calculated by subtracting the FCCP respiratory rates by the residual oxygen consumption after antimycin A) induced by different substrates in both isolated mitochondria and permeabilized flight muscle from *A*. *aegypti* females and males and then carried out correlation analyses of these values. Similar approaches were described in the literature to investigate the effect of hypoxia on mitochondrial physiology in permeabilized human skeletal muscle fibers during exercise [52, 58]. S7 Fig. shows the correlation analyses carried out for female (S7A–C Fig.) and male (S7D–F Fig.) isolated mitochondria using Pyr+Pro (S7A and S7D Fig.), G3P (S7B and S7E Fig.) and PC+Mal (S7C and S7F Fig.) as substrates. We observed that in all cases, there is a linear correlation between the OXPHOS and maximum uncoupled respiratory rates, which varied in a direct proportion, indicating preserved coupling between these two distinct mitochondrial metabolic states regardless the substrate utilized and sex. We also conducted the same analyses on permeabilized flight muscle of both sexes using Pyr+Pro (S7G and S7I Fig.) and G3P (S7H and S7J Fig.) and a pattern very similar to that of isolated mitochondria was observed. A closer look on these data revealed interesting features of mitochondrial functionality in *A*. *aegypti* flight muscle. Table 5 shows the compiled bioenergetic efficiency (slope) and capacity (OXPHOS) values of female mitochondria for all comparisons among the substrates and preparations. First, the bioenergetic capacities are remarkably distinct among the substrates (as previously shown in Tables 3 and 4), from the lowest values with PC+Mal to the highest with Pyr+Pro, indicating that the extent of substrate oxidation play a central role to bioenergetic capacity. Second, the bioenergetic efficiency, determined as the slope of correlation analyses, is high, ranging from 0.46 (G3P) to 0.74 (Pyr+Pro) both in permeabilized flight muscle (Table 5). These data suggest that bioenergetic capacity among the substrates is essentially driven by their potential to be oxidized by the mitochondrial dehydrogenases and the electron transport system complex activities, and not by the degree of mitochondrial inner membrane permeability, since the bioenergetic efficiency (slope) is roughly preserved. In males, a very similar pattern of bioenergetic efficiency and capacity among substrates and preparations was achieved (S7D-F, S7I, S7J Fig. and S4 Table). The biological significance of these findings is that, regardless the sexual differences of *A*. *aegypti*, substrates oxidation provided by mitochondrial dehydrogenases and the electron transport system activity define the bioenergetic capacity, but not efficiency as the degree of mitochondrial inner membrane permeability is preserved. Also, these data strongly suggest that proline and glucose metabolism represent the main energy-providing pathways to sustain flight in this insect [13, 28, 34, 47, 67].

**Table 5:** Mitochondrial bioenergetic efficiency and capacity in *A*. *aegypti* females flight muscle using different substrates.

**Isolated mitochondria**				
	**Bioenergetic efficiency (Slope)**	**Correlation coefficient**	***p***	**Bioenergetic capacity (OXPHOS)**
**Pyr+Pro**	0.67 ± 0.17	0.78 ^b^	0.004	118 ± 57 *
**G3P**	0.55 ± 0.08	0.77 ^a^	0.0002	39 ± 25 **
**PC+Mal**	0.72 ± 0.23	0.79 ^b^	0.021	4 ± 1
**Flight muscle**				
**Pyr+Pro**	0.74 ± 0.14	0.83 ^b^	0.0004	223 ± 45^#^
**G3P**	0.46 ± 0.06	0.84 ^b^	< 0.0001	59 ± 27

Values of bioenergetic efficiency (slope) were expressed as mean ± SD of OXPHOS versus maximum uncoupled respiratory rate linear regression and correlation analyses made in S7 Fig. Correlation coefficient values (Spearman or Pearson) were depicted as superscript letters “a” or “b”, respectively. P values represent the statistical significance of linear regression slopes in each group. Bioenergetic capacity (OXPHOS) values represent the respiratory rates data induced by ADP and calculated by subtracting the ADP rates by their equivalent "leak" values shown in Table 3 (for isolated mitochondria) and Table 4 (for flight muscle). Statistical analyses on bioenergetic capacity in isolated mitochondria were performed by using Mann Whitney test (indicated by superscript astersiks) as well as ANOVA and *a posteriori* Tukey´s test (indicated by superscript symbols) for flight muscle. Significant differences in isolated mitochondria were

* *p<0*.*005* relative to G3P and PC+Mal, and

** *p<0*.*0001* relative to PC+Mal. In flight muscle, significant difference was

^#^ *p<0*.*001* relative to G3P.

### f) Mitochondrial metabolic preference towards proline oxidation in females

**Fig 3:**
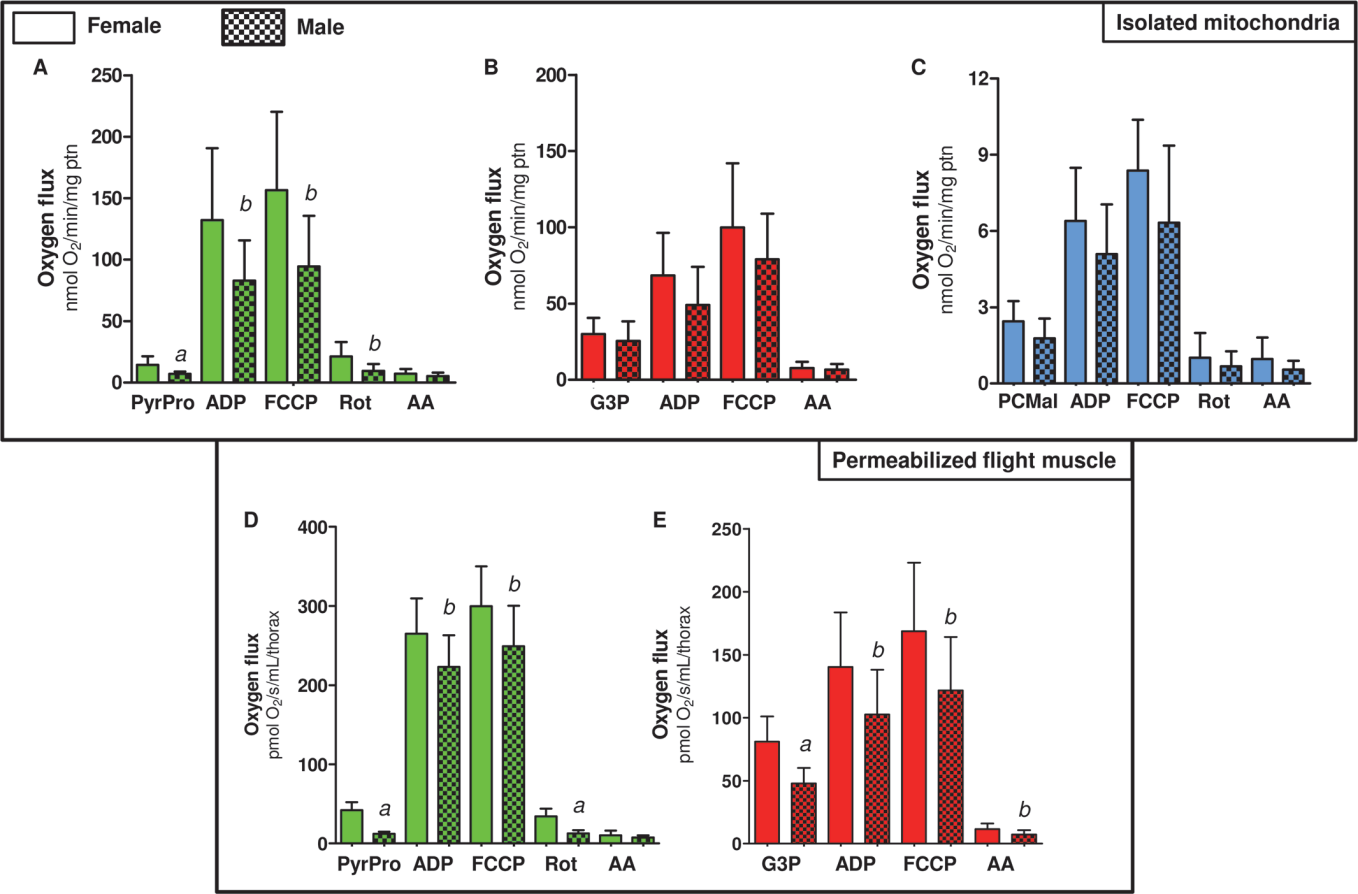
Comparative analyses of respiratory rates induced by different substrates among *A*. *aegypti* sexes. Oxygen consumption rates from isolated mitochondria (A-C) and whole permeabilized flight muscle (D and E) from females (solid bars) and males (hatched bars) were plotted from values shown in Tables 3, 4, S2 and S3. Data are expressed as mean ± SD of at least six different experiments. Comparisons between groups were done by Student´s t- test. Figure (A): ^*a*^ *p*<0.005 and ^*b*^ *p*<0.05 relative to their equivalent metabolic state in female. Figure (D): ^*a*^ *p*<0.0001 and ^*b*^ *p*<0.05 relative to their equivalent metabolic state in female. Figure (E): ^*a*^ *p*<0.001 and ^*b*^ *p*<0.05 relative to their equivalent metabolic state in female.

We next investigated whether mitochondrial oxygen consumption would be distinct among sexes in *A*. *aegypti* flight muscle. Previous reports have already addressed the sex-associated mitochondrial structural and functional differences in a variety of organisms and tissues [79–84]. Although some reports support the concept of sex-associated differences in mitochondrial function [76, 79–83], consensus about a general trend is lacking. For example, despite the levels of antioxidant defenses in rats are reduced, and redox imbalance markers are higher in males [84], mitochondrial functional parameters were quite similar in three mice tissues regardless the sex [83]. In *Drosophila* males, the rate of mitochondrial H_2_O_2_ formation is significantly lower than in females, which is consistent with higher antioxidant enzyme activities (catalase, Mn-SOD and Cu/Zn-SOD) in males [85]. In addition, OXPHOS efficiency and proton leak were higher in females compared to males, indicating that mitochondrial functional differences among sexes may reflect the increased energy demand posed by sex-specific activities, such as reproduction in females. In *A*. *aegypti* flight muscle, we observed a general trend of lower respiratory rates in males compared to females, in all substrates tested (Fig. 3). However, significantly reduced respiratory rates in males were only observed when using Pyr+pro (Fig. 3A and 3D) and G3P (Fig. 3E). Respiratory rates of male isolated mitochondria using Pyr+pro were about 38% lower than in females, while in G3P and PC+Mal these rates were about 22% lower compared to females. Interestingly, we observed that respiratory rates of female permeabilized flight muscle were significantly higher than in males, using both Pyr+pro (Fig. 3D) or G3P (Fig. 3E) as substrates.

**Fig 4:**
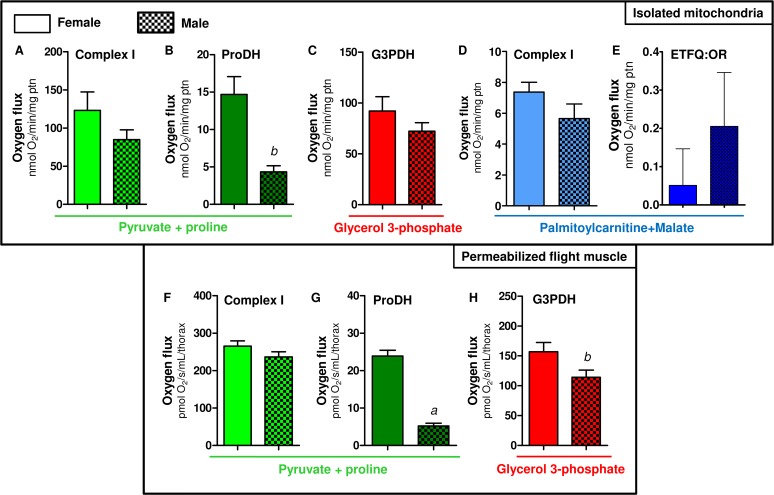
Preference towards proline oxidation in *A*. *aegypti* female mitochondria. Oxygen consumption rates from isolated mitochondria (A-E) and whole permeabilized flight muscle (F-H) from females (solid bars) and males (hatched bars) were calculated from values shown in Fig. 3. Data are expressed as mean ± SD of at least seven different experiments. Comparisons between groups were done by Student´s t tests. Figure (B): ^*b*^ *p*<0.001 relative to female; Figure (G): ^*a*^ *p*<0.0001 relative to female; Figure (H): ^*b*^ *p*<0.038 relative to female.

A closer look on the data shown in Fig. 3 reveal that the contribution of proline oxidation to respiration was strikingly higher in females. The respiratory rates induced by Pyr+pro in both isolated mitochondria (Fig. 3A) and permeabilized flight muscle (Fig. 3D) were significantly higher in females than in males (2.06 and 3.5 times higher than males, respectively). In addition, the rotenone-resistant respiration was higher in females (2.2 and 2.7 times higher than males, respectively) in both preparations (Fig. 3A and 3D). Indeed, assignment of mitochondrial dehydrogenases that provide electrons to the electron transport system (Fig. 4) indicated that respiration associated to ProDH activity was 3.4 times higher in female isolated mitochondria compared to males (Fig. 4B). This effect was specific to the respiration associated to ProDH since there were no apparent differences among the sexes in all three other sites of electron transport system (complex I, G3PDH and ETF:QOR)(Fig. 4A,4C,4D and 4E). A very similar trend was observed when the contribution of different dehydrogenases was assessed on permeabilized flight muscle (Fig. 4F-H). Noteworthy is the specifically high contribution of proline oxidation to respiration in female mitochondria (Fig. 4G), which was about 4.6 times higher than in males. Since blood feeding habit in *A*. *aegypti* is restricted to females, it seems that proline metabolism would play a key role in female physiology, as the increment of respiratory rates induced by this aminoacid is higher in obligatory blood feeding Diptera when compared to other facultative blood-suckers and even to non hematophagous insects [25]. Increased contribution of proline oxidation to respiration observed in obligatory blood-sucking insects occurs in detriment of pyruvate and G3P oxidation [25], suggesting a trend that a continuous source of dietary protein provided by the blood may have adapted flight muscle mitochondria to utilize proline as the main source of electrons to sustain respiration. In fact, flight-induced reductions in proline levels in the hemolymph and thorax of male *A*. *aegypti* were much less pronounced than observed in females [13], indicating that oxidation of this aminoacid in flight muscle is more intense in females. Conceivably, proline transport across inner mitochondrial membrane in males would be limited, directly affecting respiration, since there were no changes in proline-induced cytochrome *c* reduction among sexes (Table 2). In this sense, although in the present work mitochondrial function was assessed only in sugar fed insects, it seems possible that substrates preferences may change after blood ingestion, due to the higher protein content in this dietary source. The relatively high respiratory rates induced by G3P in *A*. *aegypti* flight muscle (Fig. 4C and 4H) suggest increased dependence of this tissue to glucose utilization, as in insect´s flight muscle the cytosolic isoform of G3PDH is largely responsible for the re-oxidation of extramitochondrial glycolysis-derived NADH instead of classical lactate dehydrogenase [86,87]. However, despite the advantages of assessing mitochondrial physiology on permeabilized flight muscle, we are aware about the potential limitations with this approach in terms of the respiratory capacities among sexes, since a clear sexual size dimorphism exists in *A*. *aegypti* (S1 Table). In this regard, correlating the uncoupled respiratory rates of whole flight muscle from individual insects of both sexes using Pyr+pro as substrates, by their respective whole body weight, gives a linear positive relationship (S8 Fig.). This indicates that permeabilized flight muscle respiration varies in a direct proportion with the insect mass, regardless the sex. Since body weight in females is higher than in males (S1 Table), this could explain the differences in respiratory rates observed in permeabilized flight muscle among sexes. We think that this would be the case for the sexual comparisons of absolute values observed for most metabolic states in permeabilized flight muscle (Figs. 3E,4F, and 4H), but not for the ProDH-dependent respiration (Figs. 3D and 4G). Indeed, the sexual differences in respiratory rates provided specifically by ProDH (Fig. 4G, females 4.6 times higher than males) overpass the predicted sexual difference in respiratory rates due only to the weight (S1 Table, females 1.7 times higher than males). Therefore, we conclude that there is a clear preference towards proline oxidation in female flight muscle mitochondria compared to males. The biological significance of these data is that specific reduced proline oxidation and respiratory rates observed in male flight muscle may explain their limited flight capacity in nature when compared to females [88].

### g) An unique mitochondrial redox feature: H_2_O_2_ production is essentially driven by pyruvate+proline and G3P oxidation, with equivalent contribution among these substrates

The contribution of mitochondria to cell physiology goes far beyond their classical role in energy metabolism, directly participating in cell signaling, apoptosis, and on redox homeostasis, representing an important source of ROS. Indeed, superoxide (O_2_^•¯^) is the primary ROS produced in at least ten different sites in mitochondria (see purple arrows in Fig. 1 and refs [29, 31, 37, 89–93]). Few studies were dedicated to understand the contribution of mitochondria to cellular ROS production in insects, despite their implication in a myriad of biological processes in these organisms including aging [12, 94–98], development [99], hypoxia tolerance [100], apoptosis [101], muscular and neuronal function [102], ecdysteroid synthesis [103], as well as on immune response [44–46]. Regarding the mechanisms involved on mitochondrial ROS generation in insects, early work conducted in *Musca domestica* flight muscle demonstrated that hydrogen peroxide (H_2_O_2_) generation was mostly supported by G3P (16-times higher) than Pyr+pro oxidation [94]. Later, Miwa and co-workers have showed that in *Drosophila* flight muscle mitochondria H_2_O_2_ formation occurred mainly by G3PDH and the center *o* of complex III (12-times higher), followed by complex I ROS generation to the mitochondrial matrix side (see Table 2 of ref [29]). Recent evidence also point out that mitochondrial proline oxidation through ProDH contributes to indirect O_2_^•¯^ production particularly at complexes I and II [37]. Given the relevance of mitochondrial redox metabolism on insect physiology, in Table 6 we compiled the mitochondrial H_2_O_2_ production data available on insects and compared with vertebrate muscle mitochondria [12, 29, 31, 90, 94–98, 101–106]. Interestingly, general trends can be pointed out here, such as: *i)* the rates of H_2_O_2_ formation supported by G3P were strikingly higher than by complex I substrates, regardless the species; *ii)* H_2_O_2_ production rates by G3P in insects are much higher than in vertebrate skeletal muscle, but close to the rates reported for heart mitochondria. Considering that reported respiratory rates induced by G3P in *Drosophila* are 52,7% lower than those produced by Pyr+pro oxidation (Table 1), while H_2_O_2_ production rates by G3P are 567% higher than by complex I (Table 6), it seems plausible that electron flow through G3PDH is more "leaky" than by complex I, resulting in increased O_2_^•¯^ formation at both sides of mitochondrial inner membrane [12, 29, 31, 90, 94–98, 101–106]. In fact, the contribution of different substrates to mitochondrial H_2_O_2_ formation has been investigated for decades and the intrinsic complexity of the system makes the determination of absolute values highly variable, depending on many parameters such as the organism model, the experimental conditions, substrates transport and oxidation, the ΔѰ_m_ magnitude, and mitochondrial dynamics [104, 107–110].

**Table 6:** Comparison of mitochondrial H_2_O_2_ production by insect flight muscle and vertebrate muscles.

	NADH-dependent	G3P-dependent
**Fruitfly *(Drosophila)*** ^[^ ^12^ ^,^ ^29^ ^,^ ^31^ ^,^ ^96^ ^]^	265	1503
**Housefly *(Musca)*** ^[^ ^95^ ^–^ ^98^ ^]^	70	1195
**Fleshfly *(Sarcophaga)*** ^[^ ^97^ ^]^	-	1950
**Blowfly *(Calliphora)*** ^[^ ^97^ ^]^	-	2150
**Vertebrate heart** ^[^ ^90^ ^,^ ^104^ ^–^ ^106^ ^]^	158	1687
**Vertebrate skeletal muscle** ^[^ ^104^ ^,^ ^106^ ^]^	165	590

Mitochondrial hydrogen peroxide formation was expressed as pmol of H_2_O_2_/min/mg protein during non-phosphorylating conditions. In the case of *Drosophila*, *Musca* and vertebrate muscles, in which multiple articles were utilized for calculations, an average value was obtained. Superscript letters indicate the data source.

Our group have previously shown that blood feeding promoted not only mitochondrial fusion in *A*. *aegypti* flight muscle, but also a drastic reduction in cytochrome *c* oxidase activity, mitochondrial oxygen consumption and H_2_O_2_ formation [34]. We speculated that functional and structural mitochondrial remodeling upon blood feeding would avoid the interaction of mitochondrial-derived ROS with blood derived products, such as heme and iron, which could lead to redox imbalance and eventually tissue damage [34, 102, 111]. Thus, in order to gain a deeper insight on the contribution of mitochondria to *A*. *aegypti* redox metabolism, we assessed the H_2_O_2_ production on isolated flight muscle mitochondria. S4 Fig. show representative fluorimetric traces of H_2_O_2_ formation by male and female mitochondria from *A*. *aegypti* flight muscle using Pyr+pro as substrates. H_2_O_2_ generation rates during phosphorylating conditions proceeded stably, which were boosted after F_1_F_o_ ATP synthase inhibition by oligomycin, and subsequently reduced by OXPHOS uncoupling promoted by the proton ionophore FCCP. Then, for Pyr+pro and PC+Mal, complex I inhibitor rotenone was added, causing small increases on H_2_O_2_ generation rates, which were further increased upon complex III inhibition by antimycin A, and resulting in the maximal rates of H_2_O_2_ production [108, 112]. When using G3P as substrate, experiments started by the addition of 0.5 μM rotenone. Table 7 shows that mitochondrial H_2_O_2_ formation rates in both sexes were in general 20–80% higher during non-phosphorylating conditions (oligomycin) than after uncoupling by FCCP. However, these differences were only significant when using G3P (on both sexes) or PC+Mal (only females), indicating their higher dependence on ΔѰ_m_ to generate O_2_^•¯^. This contrasts to data obtained with mice skeletal muscle mitochondria oxidizing long chain fatty acids, which revealed to be only slightly affected by reductions in the ΔѰ_m_ [113]. Mitochondrial H_2_O_2_ production rates in *A*. *aegypti* flight muscle induced by Pyr+pro or G3P during non-phosphorylating (oligomycin) conditions were indistinguishable among each other, and significantly higher than with PC+Mal (Table 7). Indeed, a comprehensive investigation in different rat tissues revealed that fatty acid oxidation plays a major role in mitochondrial H_2_O_2_ production only in kidney and liver [104]. These data indicate that fatty acid oxidation plays a minor role on cytochrome *c* reduction (Table 1), respiration (Tables 3, S2, S4 Figs., Figs. S5A, S7C, S7F, 2A, 3C, 4D, 4E) and H_2_O_2_ formation (Table 7) in *A*. *aegypti* flight muscle mitochondria. Comparative analyses between Tables 6 and 7 show that NADH-dependent H_2_O_2_ generation in *A*. *aegypti* mitochondria is higher than any other organism (about 49% and 464% higher than *Drosophila* and *Musca*, respectively). Considering that the respiratory rates mediated by complex I substrates in *A*. *aegypti* flight muscle were lower than in other insects species (Tables 1, 3 and S2), we suggest that redox reactions that provide NADH to complex I such as pyruvate dehydrogenase and α-ketoglutarate dehydrogenase, might be involved in controlling both respiration and H_2_O_2_ generation. On the other hand, mitochondrial H_2_O_2_ generation rates in *A*. *aegypti* using G3P were strikingly lower when compared to other insects (80% and 72% lower than *Calliphora* and *Drosophila*, respectively) and even to vertebrate mitochondria (28% and 75% lower than skeletal muscle and heart, respectively). In *Drosophila* mitochondria, G3P-induced H_2_O_2_ production was 7.6 times higher than with Pyr+Pro (see Table 2 on ref [29]), and was also less ΔѰ_m_ dependent than complex I [29]. Interestingly, as previously pointed out (Tables 1, 3 and S2), G3P-induced respiration in *A*. *aegypti* was lower than all insect species and even vertebrates (44% and 81% lower than *Popillia* and *Drosophila*, respectively). A plausible explanation for these data is that mitochondrial G3PDH activity in *A*. *aegypti* may be lower than in any other organism, which would limit electrons entry at the electron transport system and keeping both oxygen consumption and H_2_O_2_ production rates reduced. Together, we conclude that Pyr+pro and G3P are the main substrates that drive mitochondrial H_2_O_2_ production in *A*. *aegypti* flight muscle mitochondria, which were both equivalent in terms of their capacity to generate ROS (Table 7). This feature, instead of the usual preference towards G3P oxidation to produce H_2_O_2_ (Tables 6 and 7), represents a unique mitochondrial redox feature present in *A*. *aegypti* flight muscle.

**Table 7:** Contribution of different substrates to mitochondrial H_2_O_2_ production in *A*. *aegypti* flight muscle.

**Female**						
**Modulator**	**Pyr+Pro**	n	**G3P**	n	**PC+Mal**	n
Oligomycin	371 ± 112	5	369 ± 82 **	9	112 ± 28 ^*a*^ ^,^ ^#^	5
+ FCCP	207 ± 104	5	235 ± 42	9	66 ± 23 ^*c*^	5
**Male**						
**Modulator**	**Pyr+Pro**	n	**G3P**	n	**PC+Mal**	n
Oligomycin	419 ± 94	5	473 ± 62 *	9	128 ± 19 ^*b*^	5
+ FCCP	271 ± 111	5	396 ± 61	9	98 ± 19 ^*d*^	5

Values were expressed as mean ± SD of pmol hydrogen peroxide produced/min/mg protein in two different mitochondrial metabolic states using: 10 mM pyruvate + 10 mM proline (Pyr+pro), 20 mM *sn* glycerol-3 phosphate (G3P), or 10 μM palmitoylcarnitine + 5 mM malate (PC+Mal) followed by 2 mM ADP, 4 μg/mL oligomycin (Oligo), and 2 μM FCCP. For all G3P experiments, measurements were carried out after addition of 0.5 μM rotenone. Statistical analyses were carried out between the groups of different substrates and mitochondrial metabolic state within the same sex, and were performed by using Kruskal-Wallis test followed by *a posteriori* Dunn´s test (indicated by superscript letters). Significant difference in “Oligo” was

^*a*^ *p = 0*.*0052*, relative to Pyr+pro and G3P,

^*b*^ *p = 0*.*0045*, relative to G3P. Significant differences in “FCCP” was;

^*c*^ *p = 0*.*0065*, relative to G3P;;

^*d*^ *p = 0*.*0016*, relative to G3P. Analyses were also conducted between the two mitochondrial metabolic states (oligo vs. FCCP) within the same substrate and sex and were performed by using Mann Whitney test (indicated by superscript symbols). Significant differences in G3P were ** *p*<0.0001 relative to female FCCP;

* *p* = 0.016 relative to male FCCP. Significant difference in PC+Mal were

# *p* = 0.03 relative to female FCCP;

### h) The major sites of superoxide production mediated by proline and G3P oxidation takes place at distinct points other than site I_F_

Considering that mitochondrial electron leak take place in at least ten different sites [29, 31, 37, 89–91, 93, 104, 106, 112–114]), we next investigated the topology of H_2_O_2_ production in *A*. *aegypti* flight muscle mitochondria. Table 8 shows the effects of rotenone, and subsequently antimycin A, on H_2_O_2_ generation in mitochondria using Pyr+pro, G3P and PC+Mal under uncoupled (FCCP) conditions. This approach has some advantages in terms of assessing mitochondrial redox metabolism, since uncoupling keep the electron transport system enzymes in a state that would not be inhibited by their products, as well as to consume NAD(P)H, which lowers the transhydrogenase activity and then the pool of reduced glutathione. A general trend observed is that inhibition of complex I by rotenone caused only slight increases (about 25%) on Pyr+pro-induced H_2_O_2_ formation, regardless the sex. It is known that complex I produce O_2_^•¯^ at I_F_ and I_Q_ sites [114], which are stimulated by increased NADH/NAD^+^ ratio and high protonmotive force (pmf), respectively. Since O_2_^•¯^ generation at site I_Q_ requires high pmf to allow reverse electron transfer to complex I, and in Table 8 all measurements were conducted in the presence of FCCP, we conclude that induction of H_2_O_2_ generation caused by rotenone can only be accounted by the I_F_ site of complex I. Significant increases in rotenone-induced H_2_O_2_ generation were only achieved in female mitochondria when using PC+Mal as substrates (57% higher than FCCP) (Table 8). Notwithstanding, Pyr+Pro and G3P H_2_O_2_ production rates in the presence of rotenone were indistinguishable, but significantly higher than in PC+Mal in both sexes. When antimycin A, which inhibits center *i* of complex III, was added subsequently to rotenone, a strong induction of H_2_O_2_ formation was achieved (Table 8). However, this increase was not homogeneous among substrates, since the absolute rates of mitochondrial H_2_O_2_ generation in this condition varied from 769 pmol H_2_O_2_/min/mg protein using Pyr+pro to about 173 pmol H_2_O_2_/min/mg protein by PC+Mal. Since the electron entry at electron transport system by complex I is inhibited by rotenone, we reasoned that the only available electron sources would be through alternative dehydrogenases such as G3PDH, ProDH and ETF:QOR. In this sense, we observed that after antimycin A, H_2_O_2_ generation rates induced by Pyr+pro increased about 170% over the rates produced by rotenone alone, indicating that redox reactions involved in mitochondrial proline oxidation, through ProDH and other dehydrogenases [37], play a major role in mitochondrial H_2_O_2_ formation in *A*. *aegypti* flight muscle mitochondria. Antimycin A also caused a drastic increase on G3P-mediated mitochondrial H_2_O_2_ generation (about 111% higher than rotenone in both sexes) (Table 8), which could be assigned to G3PDH and other dehydrogenases as well [90]. Finally, inhibition of complex III when mitochondria oxidize PC+Mal also increased H_2_O_2_ generation rates in about 38% compared to the rates on rotenone in both sexes, although with absolute values significantly lower than obtained by Pyr+pro and G3P. These data are in full agreement with the literature showing that inhibition of complex III in skeletal muscle mitochondria respiring PC increased H_2_O_2_ generation rates about 6 times compared to rates induced by rotenone [113].

**Table 8:** Topology of H_2_O_2_ formation in *A*. *aegypti* flight muscle mitochondria.

**Female**						
**Modulator**	**Pyr+Pro**	n	**G3P**	n	**PC+Mal**	n
FCCP	207 ± 104	5	-	-	66 ± 23	5
+ Rotenone	260 ± 94	5	235 ± 42	9	104 ± 31 ^●^	5
+ Antimycin	685 ± 236 ^*a*^	5	538 ± 99 *	9	155 ± 45 ^*c*^ ^,^ ^□^	5
**Male**						
**Modulator**	**Pyr+Pro**	n	**G3P**	n	**PC+Mal**	n
FCCP	271 ± 111	5	-	-	98 ± 19	5
+ Rotenone	310 ± 75	5	396 ± 61	9	110 ± 13 ^●^	5
+ Antimycin	855 ± 159 ^*b*^	5	770 ± 186 ^#^	9	192 ± 53 ^*d*^ ^,^ ^□^	5

Values were expressed as mean ± SD of pmol hydrogen peroxide produced/min/mg protein in three different mitochondrial metabolic states using: 10 mM pyruvate + 10 mM proline (Pyr+pro), 20 mM *sn* glycerol-3 phosphate (G3P) or 10 μM palmitoylcarnitine + 5 mM malate (PC+Mal) followed by 2 mM ADP, 4 μg/mL oligomycin, 2 μM FCCP, 0.5 μM rotenone, 2.5 μg/mL antimycin A. Statistical analyses within groups were performed by using Mann Whitney test (superscript letters), Student t test (superscript symbols) or Kruskal-Wallis test followed by *a posteriori* Dunn´s test (superscript open squares, □ or closed circles ^●^).

^a^, p = 0.0079, relative to female Pyr+pro FCCP;

^b^, p = 0.0079, relative to male Pyr+pro FCCP;

^c^, p = 0.0079, relative to female PC+Mal FCCP;

^d^, p = 0.03, relative to male PC+Mal FCCP;

*, p<0.0001 relative to female G3P Rot;

#, p<0.0001 relative to male G3P Rot.

^□^, p = 0.004, relative to male and female Pyr+pro and G3P antimycin.

^●^, p = 0.005, relative to female Pyr+pro and G3P and male G3P antimycin.

**Fig 5:**
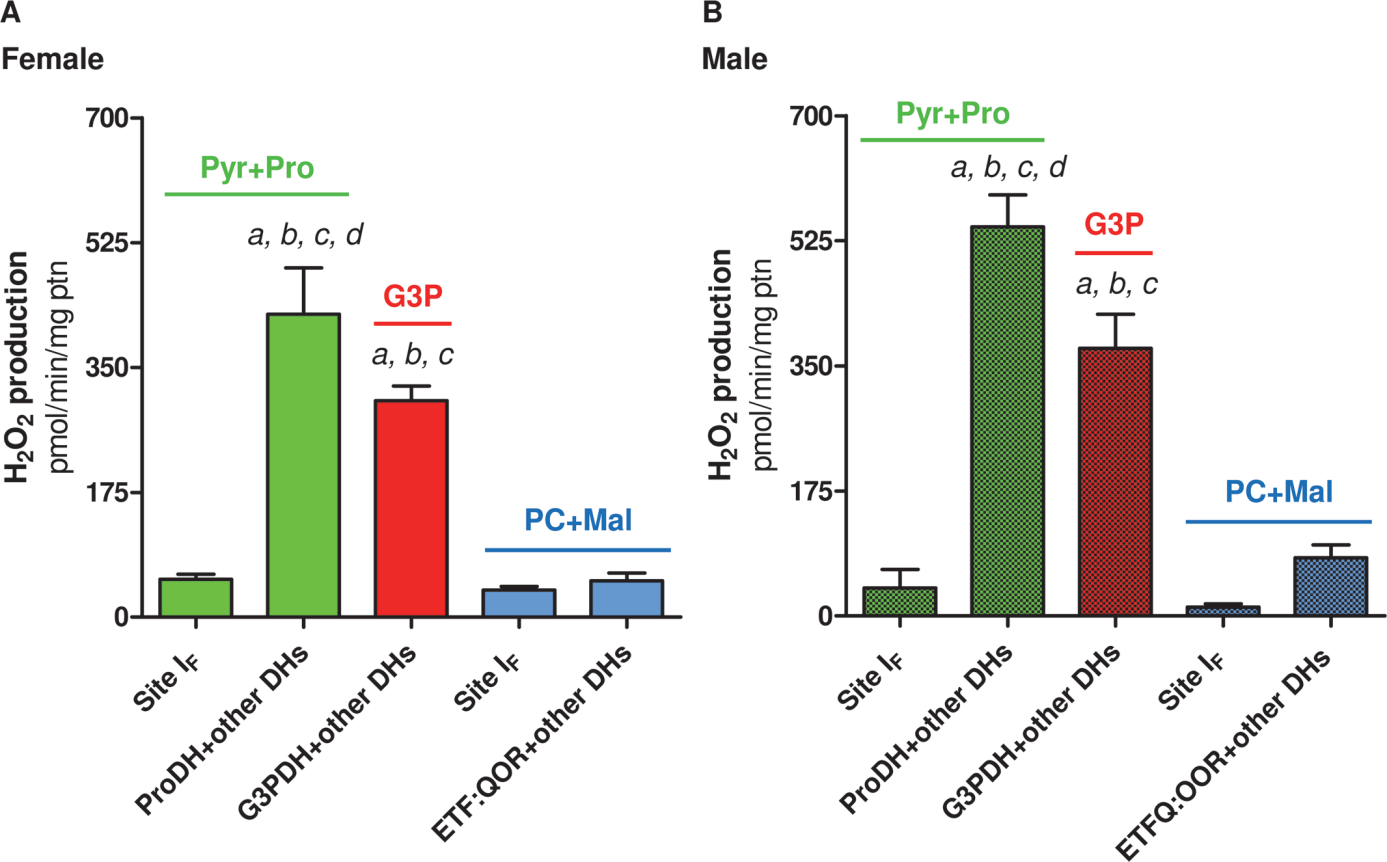
Contribution of different electron leak sites to H_2_O_2_ generation in isolated *A*. *aegypti* flight muscle mitochondria. The contribution of site I_F_, ProDH+other dehydrogenases, G3PDH+other dehydrogenases and ETF:QOR+other dehydrogenases sites to H_2_O_2_ generation in *A*. *aegypti* mitochondria isolated from females (A, solid colors) and males (B, hatched bars) were calculated from data shown in Table 8. Data are expressed as mean ± SD of at least five different experiments. Comparisons between groups were done by ANOVA and *a posteriori* Tukey´s tests. Figure (A): ^*a*^ *p*<0.001 relative to I_F_ (Pyr+pro); ^*b*^ *p*<0.001 relative to ETF:QOR+other dehydrogenases; ^*c*^ *p*<0.001 relative to I_F_ (PC+Mal); ^*d*^ *p*<0.05 relative to G3PDH+other dehydrogenases; Figure (B): ^*a*^ *p*<0.001 relative to I_F_ (Pyr+pro); ^*b*^ *p*<0.001 relative to ETF:QOR+other dehydrogenases; ^*c*^ *p*<0.001 relative to I_F_ (PC+Mal); ^*d*^ *p*<0.05 relative to G3PDH+other dehydrogenases.

Fig. 5 shows the contribution of the accountable sites of electron leak to H_2_O_2_ generation in *A*. *aegypti* flight muscle mitochondria, using the data generated by the experiments shown in Table 8. We observe that the pathways involved in proline and G3P oxidation are, by far, the most important electron leak sites to support H_2_O_2_ generation in *A*. *aegypti* flight muscle mitochondria, regardless the sex. When *A*. *aegypti* flight muscle mitochondria utilize G3P as a substrate, O_2_^•¯^ formation can be attributed to G3PDH and/or other dehydrogenases, since all experiments with this substrate were carried out in the presence of rotenone. Therefore, despite the exact site of electron leak on *A*. *aegypti* mitochondria respiring G3P could not be assigned, at least the contribution of site I_F_ to O_2_^•¯^ formation can be ruled out (Fig. 5). We must also consider that *A*. *aegypti* females have basically two distinct dietary sources (sugar and blood), which, by means of proline (from blood) or glucose (from sap) metabolism, may significantly contribute to mitochondrial O_2_^•¯¯^ formation.

Mitochondrial proline oxidation in *Drosophila* flight muscle mediates H_2_O_2_ production essentially by means electron leak at sites II_F_ (∼70%) and I_F_, with no direct contribution by either ProDH and α-ketoglutarate dehydrogenase activities [37]. Since proline metabolism play a central energetic role to sustain flight activity in *A*. *aegypti* [13], mitochondrial oxidation of this aminoacid may have important implications not only to cellular redox homeostasis but also to insect physiology. The huge increase on H_2_O_2_ production rates provided by antimycin A takes place essentially at ProDH and/or other dehydrogenases, but not at site I_F_ as rotenone was present during this assay (Fig. 5). Therefore, based on the literature [37, 90, 113], it seems plausible that site II_F_ would play a significant role in providing electrons to allow O_2_^•¯^ production in *A*. *aegypti* flight muscle mitochondria when oxidizing proline or G3P. Regarding H_2_O_2_ generation driven by fatty acid oxidation, the increases provided by complex III inhibition occurs by ETF:QOR+other dehydrogenases but also at site I_F_ in similar proportion, specifically in females (Fig. 5A, blue bars). Notwithstanding, in male mitochondria (Fig. 5B), H_2_O_2_ generation at site I_F_ is significantly lower than at ETF:QOR+other dehydrogenases sites, which is in agreement with data obtained for skeletal muscle oxidizing PC [113]. Indeed, the small contribution of fatty acid oxidation to O_2_^•¯^ formation can be just a consequence of reduced metabolism of this nutrient, based on the very low cytochrome *c* reductase activity induced by PC in *A*. *aegypti* mitochondria (Table 2). Thus, the major sites of O_2_^•¯^ production in *A*. *aegypti* flight muscle mitochondria during proline and G3P oxidation takes place at distinct points other than site I_F_.

Finally, we must consider the potential limitations in determining the sites of O_2_^•¯^ generation in our experimental approach since *i)* the use of electron transport system inhibitors does not allow the measurements of native H_2_O_2_ production rates relative to different substrates and sexes [54]; *ii)* as a consequence, we cannot assign precisely the sites of O_2_^•¯^ formation, since in the presence of rotenone and antimycin A, electrons coming from substrate oxidation leak at many different sites [37, 54, 90]. Also, we are aware that changes in NADPH levels and matrix manganese superoxide dismutase (SOD2) activity may represent important sources of interference in our measurements. However, we don´t think these are the cases, since: *i)* all determinations were carried out based on standard curves built with H_2_O_2_ in the presence of each pharmacological OXPHOS modulator and 0.17 mg of mitochondrial protein, which, in principle, would correct for eventual changes in NADPH levels; *ii)* changes on mitochondrial O_2_^•¯^ production by OXPHOS modulators affect targets upstream of SOD2 and it is unlikely that the pattern of H_2_O_2_ production observed in our experiments (S4 Fig.) would be resulted from changes on SOD2 activity. Despite these limitations, the partial assignment of O_2_^•¯^ production sites in *A*. *aegypti* mitochondria contribute to a broader knowledge of mitochondrial redox metabolism in this organism, with potential physiological implications.

### i) Increased H_2_O_2_ generation in A. aegypti male mitochondria occurs specifically by means of G3P oxidation

**Fig 6:**
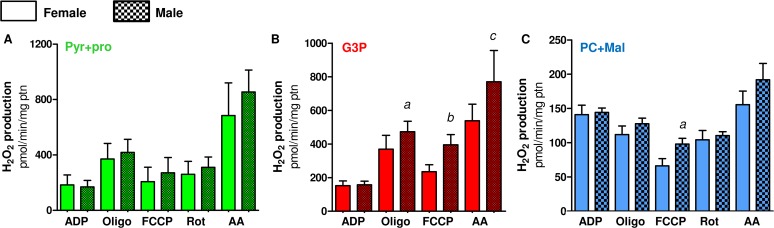
Comparative analyses of H_2_O_2_ generation rates induced by different substrates among *A*. *aegypti* sexes. H_2_O_2_ formation rates of mitochondria isolated from females (solid bars) and males (hatched bars) were plotted using the values shown in Tables 7 and 8. Data are expressed as mean ± SD of at least five different experiments. Comparisons between groups were done by Student´s t-test. Figure (B): ^*a*^ *p*<0.005, ^*b*^ *p*<0.0001 and ^*c*^ *p*<0.005 relative to their equivalent metabolic state in female. Figure (C): ^*a*^ *p*<0.05 relative to its equivalent metabolic state in female.

**Fig 7:**
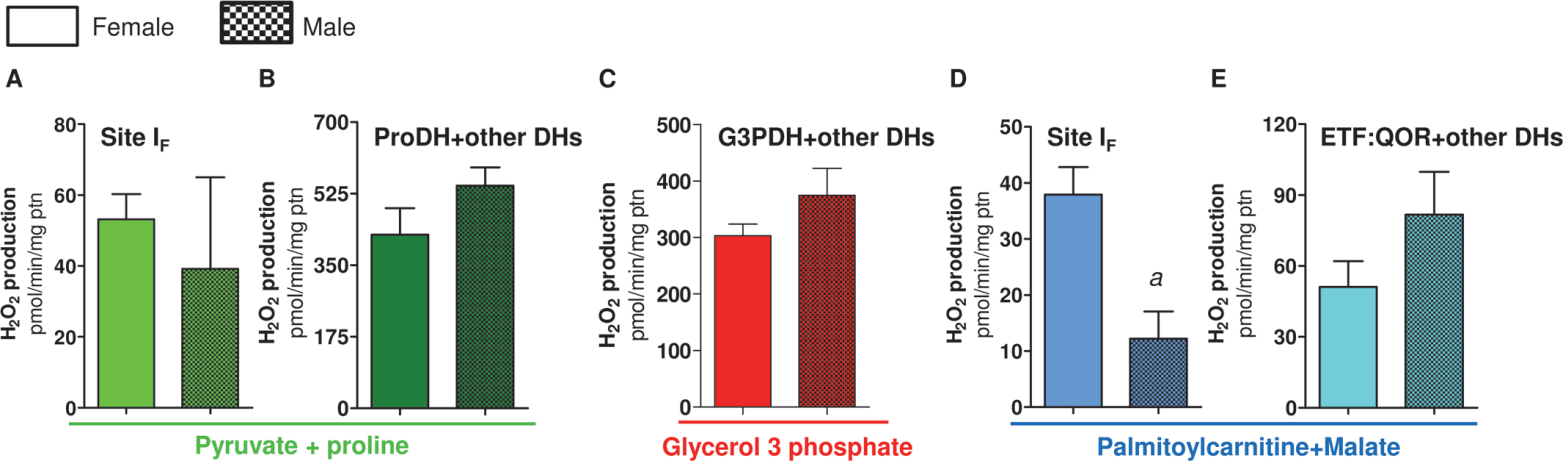
Sexual differences in the contribution of different electron leak sites to H_2_O_2_ generation in isolated *A*. *aegypti* flight muscle mitochondria. The contribution of site I_F_, ProDH+other dehydrogenases, G3PDH+other dehydrogenases and ETF:QOR+other dehydrogenases sites were calculated in *A*. *aegypti* mitochondria isolated from females (solid colors) and males (hatched bars) from data shown in Table 8. Data are expressed as mean ± SD of at least five different experiments. Comparisons between groups were done by Mann Whitney test. Figure (D): ^*a*^ *p*<0.01 relative to female I_F_ (PC+Mal).

A general trend in nature is that, in many species, females live longer than males [84,115], observations that can also be extended to most insects species including *A*. *aegypti* [116]. Interestingly, in *Drosophila sp*., this trend seems not to be followed, since females are short-lived than males [117] and mitochondrial H_2_O_2_ generation is significantly lower in males, as well as the activities of antioxidant enzymes were higher in males than in females [85]. Whether altered mitochondrial redox metabolism directly participates in the aging process or acts indirectly through ROS-dependent signaling pathways in the physiological changes of senescence it is still a debatable issue. In this sense, considering the "mitochondrial free radical theory of aging", one should predict that the long lived group would have reduced levels of mitochondrial ROS generation, oxidative molecular damage, cell and tissue dysfunction [118]. Based in this information, we then compared the rates of mitochondrial H_2_O_2_ generation in both sexes of *A*. *aegypti* flight muscle, using Pyr+pro, G3P or PC+Mal as substrates. We can observe in Fig. 6 that there were no significant differences among sexes on H_2_O_2_ generation in *A*. *aegypti* flight muscle mitochondria using Pyr+pro (Fig. 6A) or PC+Mal (Fig. 6C). However, in Fig. 6B we observed that H_2_O_2_ generation rates induced by G3P were significantly higher in male mitochondria than in females, in most mitochondrial metabolic states, with exception of the G3P+ADP. The significantly higher rates of H_2_O_2_ formation were observed in non-phosphorylating state in the presence of oligomycin (28% higher than in females), in uncoupled state (FCCP) (68% higher than in females), and after inhibition by complex III by antimycin A (43% higher than females). Despite G3P-induced H_2_O_2_ generation represent, by far, the dominant sources of ROS in mitochondria from distinct insect species (Table 6), Pyr+pro and G3P oxidation are the substrates that give, equally, the highest rates of H_2_O_2_ production in both sexes of *A*. *aegypti* (Table 7). Interestingly, increased H_2_O_2_ generation in male mitochondria (Fig. 6B) associates to reduced male lifespan, compared to females [116]. Indeed, preliminary observations from our group indicate that insects from our colony exhibited remarkable differences in longevity among sexes, where female average life span (females = 34 days *vs*. males = 19 days), as well as maximum life span (females = 56 days *vs*. males = 42 days) were significantly higher compared to males (data not shown). Regarding the topology of mitochondrial electron leak, the only significant difference observed among sexes (Fig. 7) was that females exhibited higher O_2_^•¯^ generation mediated by site I_F_, when PC+Mal compared to male H_2_O_2_ generation under the same conditions. (Fig. 7D).

### j) Conclusion and final remarks

**Fig 8:**
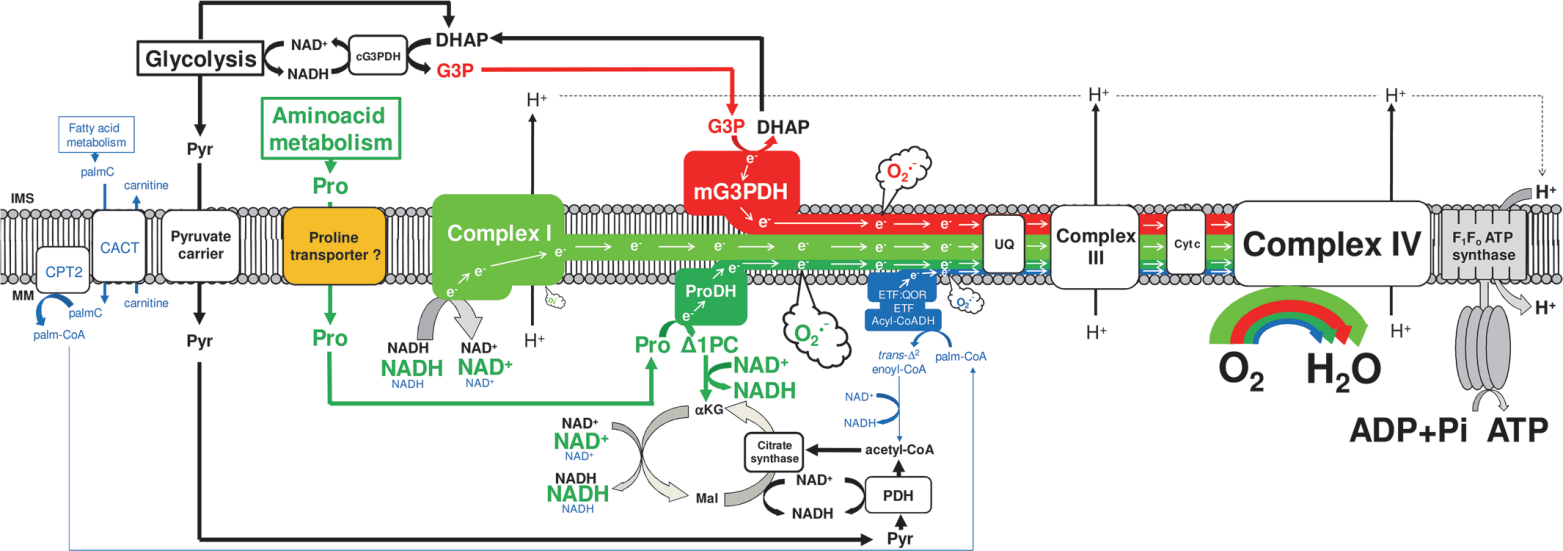
Schematic representation of substrate utilization pathways driving respiration and O2•¯ formation in *A*. *aegypti* flight muscle mitochondria. The dehydrogenases directly involved on mitochondrial electron transfer from nutrient oxidation to respiration are depicted in their respective colors utilized throughout this work, as following: complex I (light green), ProDH (dark green), G3PDH (red) and ETF:QOR (blue). The contribution of dehydrogenases to respiration are represented by their boxes, fonts, and lines sizes. Electron leak and O_2_^•¯^ formation induced by different substrates are represented by steam clouds, obeying the same color and size pattern described for dehydrogenases. Noteworthy, the steam cloud location in this scheme does not represent the exact site of O_2_^•¯^ production, since we were unable to precisely define these sites in this work. CACT, carnitine-acylcarnitine transferase; CPT2, carnitinepalmitoyl transferase 2; palm-CoA, palmitoyl-CoA; αKG, alpha-ketoglutarate; Δ1PC, Δ-1-pyrroline-5-carboxylate; DHAP, dihydroxyacetone phosphate; PDH, pyruvate dehydrogenase; IMS, intermembrane space; MM, mitochondrial matrix.

A summary of the metabolic pathways operating in *A*. *aegypti* flight muscle mitochondria described in this work is schematically depicted in Fig. 8. Complex I (light green) and G3PDH (red) represent the main sites of electron supply to electron transport system, followed by ProDH (dark green) and in a less extent ETF:QOR (blue), that contribute to respiration and O_2_^•¯^ formation. Given the key role of proline oxidation to flight activity, as well as on mitochondrial energy and redox metabolism, as demonstrated in the present work, we postulate that most of the electrons that are transferred to electron transport system through complex I originates from oxidation of this aminoacid. Interestingly, proline levels in the hemolymph of sugar-fed females are about twice of males, and their levels are halved upon 30 minutes of induced flight, only in females [13]. This indicates that mechanisms devoted to sustain high levels of proline in the hemolymph, as well as its utilization to support flight, are more efficient in females than in males [13]. Noteworthy, we know that floral nectars are utilized as a dietary source for both *A*. *aegypti* sexes in nature, which are enriched in carbohydrates and poor in aminoacids [119]. Therefore, even in a low aminoacid food source, *A*. *aegypti* females preserve the pathways to keep high proline levels, given the key importance of this aminoacid to energy and redox metabolism. Since mitochondrial transport of proline [120] and pyruvate [121] are dependent of ΔѰ_m_, it seems unlikely that reduced proline oxidation observed in male mitochondria would be related to low ΔѰ_m_, as complex I-dependent respiration is roughly similar among sexes (Fig. 4A and 4F), despite strikingly lower ProDH-dependent respiration in males (Fig. 4B and 4G). In addition, there were no sexual differences in terms of proline-mediated cytochrome *c* reduction in *A*. *aegypti* mitochondria (Table 2). As the molecular entity that mediate mitochondrial proline transport remains elusive, we postulate that reduced proline oxidation in *A*. *aegypti* male mitochondria is a result of reduced transport of this aminoacid compared to females (orange box in Fig. 8). The biological significance of improved mitochondrial proline transport in females could be to keep proline as preferential substrate to sustain flight activity, sparing glucose and lipids to support oogenesis/embryogenesis after blood intake. Thus activation of metabolic pathways directed to keep high hemolymph proline levels in females, regardless the dietary source, would represent a female-specific pre-adaption to allow flight muscle respiration. The contribution of different substrates to O_2_^•¯^ production were also proportional to their utilization, with the highest values obtained by Pyr+pro and G3P (Table 7), due to electron leak taking place at ProDH, G3PDH and other dehydrogenases sites (Table 8 and Fig. 7).

Despite the key role of mitochondria to cell physiology, and the growing need to understand basic aspects of NTD insect vectors biology aiming the development of potential new control strategies, few studies have investigated mitochondrial functionality in this group of organisms [34, 35, 43, 45–48, 61]. The systematic assessment of mitochondrial physiology in *A*. *aegypti* carried out in the present work, represents a significant step towards the understanding of basic functional processes that take place in this organelle. Future research will provide insights over key aspects described here, especially on the contribution of proline for mosquito energy and redox metabolism, and the potential implications for dispersal, reproduction, survival, aging, insecticide resistance and pathogen transmission.

## Supporting Information

S1 Fig Typical traces of oxygen consumption in isolated mitochondria using Pyr-Pro as substrate. Representative oxygen flux (solid green lines) and concentration (grey dashed lines) traces from female (A) or male (B) isolated *A*. *aegypti* flight muscle mitochondria. The substrate concentrations and OXPHOS modulators added in the experiment followed the description in the methods section. The amount of mitochondrial protein for each experiment is indicated.
(TIF)

S2 Fig Typical traces of oxygen consumption in permeabilized flight muscle using Pyr-Pro as substrate. Representative oxygen flux (solid green lines) and concentration (grey dashed lines) traces from a single flight muscle from female (A) or male (B) *A*. *aegypti*. The substrate concentrations and OXPHOS modulators added in the experiment followed the description in the methods section.
(TIF)

S3 Fig Typical traces of oxygen consumption in permeabilized flight muscle using G3P as substrate. Representative oxygen flux (solid red lines) and tension (grey dashed lines) traces of single flight muscle from female (A) or male (B) *A*. *aegypti*. The concentrations of G3P and OXPHOS modulator added in the experiment followed the description in the methods section.
(TIF)

S4 Fig Representative traces of hydrogen peroxide formation from female (thin line) and male (thick line) isolated *A*. *aegypti* flight muscle mitochondria using Pyr+pro as substrates. The arrows indicate the addition of OXPHOS modulators and the line slope define the rates of hydrogen peroxide formation as described in the methods section.
(TIF)

S5 Fig Complex I and G3PDH represent the major electron donor sites to support respiration in male *A*. *aegypti* flight muscle. Oxygen consumption rates from *A*. *aegypti* male isolated mitochondria (A) and permeabilized flight muscle (B) were calculated from values shown in S2 and S3 Tables. Data are expressed as mean ± SD of at least seven different experiments. Comparisons between groups were done by ANOVA and *a posteriori* Tukey´s tests (Figure A) or Kruskal-Wallis and *a posteriori* Dunn´s tests (Figure B). Figure (A): ^*a*^ *p*<0.05 relative to Complex I Pyr+pro; ^*b*^ *p*<0.05 relative to G3P; ^*c*^ *p*<0.01, relative to Complex I Pyr+pro; ^*d*^ *p*<0.001, relative to Complex I Pyr+pro and G3P. Figure (B): ^*a*^ *p*<0.001 relative to complex I; ^*b*^ *p*<0.05 relative to complex I and ProDH.
(TIF)

S6 Fig Correlation and linear regression analyses of maximum non-coupled respiration data obtained by permeabilized flight muscle and isolated mitochondria in *A*. *aegypti* females and males using different substrates. Comparative analyses of respiratory rates obtained by permeabilized flight muscle and isolated mitochondria from females (closed symbols) and males (open symbols) using Pyr+pro (circles) or G3P (triangles) as substrates in the presence of ADP and FCCP, using the data shown in Tables 3, S2, 4 and S3. The concentrations of each substrate or OXPHOS modulator added in the experiment followed the description in the methods section. *p*<0.0001 Pearson´s correlation analyses.
(TIF)

S7 Fig Assessment of bioenergetic efficiency and capacity in *A*. *aegypti* flight muscle using distinct substrates. Oxygen consumption rates from isolated mitochondria (A-F) and permeabilized flight muscle (G-J) from females (open symbols) and males (closed symbols) were determined using Pyr+pro, G3P and PC+Mal as substrates. OXPHOS and maximum uncoupled respiratory rate (ETS) respiratory rates of individual experiments were calculated, as described in the methods section. Then, OXPHOS respiratory rates were plotted as a function of their respective ETS rates, correlation and linear regression analyses were carried out and the obtained values for bioenergetic efficiency and capacity were expressed on Table 5 and S4.
(TIF)

S8 Fig Mitochondrial oxygen consumption in permeabilized flight muscle varies in a direct proportion with the insect body weight. The graph shows the maximum non-coupled respiration rates of thoraxes of individual insects (females and males together) with their respective body weight mass. Oxygen flux rates were obtained from Tables 4 and S3 of females and males using Pyr+pro or G3P as substrates. The concentrations of each substrate or OXPHOS modulator added in the experiment followed the description in the methods section. p = 0.0037 Pearson´s correlation analyses.
(TIF)

S1 Table Sexual size dimorphism and flight muscle mitochondrial protein yield of *A*. *aegypti*. Values were expressed as mean ± SD. Insect´s body weight was expressed as mg/insect. Data from wings length were expressed as mm and obtained from [2]. Data of wings area were expressed as mm^2^ and obtained from [55,56]. Mitochondrial protein yield was obtained from 120 insects (males or females) and was expressed as mg protein/mL of final preparations. Statistical analyses between groups were performed by using Mann-Whitney or unpaired t-test. ^*a*^ *p*<0.0001, ^*b*^ *p<0*.*007* all relative to male.
(PDF)

S2 Table Contribution of different substrates to respiration of isolated mitochondria from flight muscle of *A*. *aegypti* males. Values were expressed as mean ± SD of nmol oxygen consumed/min/mg protein with the following substrates: 10 mM pyruvate + 10 mM proline (Pyr+Pro), 20 mM *sn* glycerol-3 phosphate (G3P) or 10 μM palmitoylcarnitine + 5 mM malate (PC+Mal). Addition of OXPHOS modulators were indicated as "+" in the first column as following: 2 mM ADP (+ADP), 10 μM cytochrome c (not shown), 2 μM FCCP (+FCCP), 0.5 μM rotenone (+Rotenone), and finally 2.5 μg/mL antimycin A (+ Antimycin). For all G3P measurements, experiments started after the addition of 0.5 μM rotenone. Statistical analyses were carried out only between the groups of different substrates and mitochondrial metabolic state and were performed by using either Kruskal-Wallis test followed by *a posteriori* Dunn´s test (indicated by superscript letters) or by ANOVA and *a posteriori* Tukey´s test (indicated by superscript symbols). Significant differences in “Leak” were ** *p<0*.*001* relative to Pyr+Pro and PC+Mal. In “ADP”, significant differences were * *p<0*.*05* relative to Pyr+Pro, ^#^ *p<0*.*001* relative to PC+Mal. In “FCCP”, significant differences were # *p<0*.*001* relative to PC+Mal.
(PDF)

S3 Table Contribution of different substrates to sustain respiration on permeabilized flight muscle of *A*. *aegypti* males. Values were expressed as mean ± SD of pmol O_2_/s/mL/thorax in five different mitochondrial metabolic states using: 10 mM pyruvate + 10 mM proline, 20 mM *sn* glycerol-3 phosphate, followed by the addition of 2 mM ADP (ADP), 10 μM cytochrome c (not shown), 2.5 μM FCCP, 0.5 μM rotenone, 2.5 μg/mL antimycin A. Statistical analyses were performed using Mann-Whitney test. ^*a*^ *p<0*.*001* relative to Pyr+Pro.
(PDF)

S4 Table Mitochondrial bioenergetic efficiency and capacity in *A*. *aegypti* males flight muscle using different substrates. Values of bioenergetic efficiency (slope) were expressed as mean ± SD of OXPHOS versus maximum uncoupled respiratory rate linear regression and correlation analyses made in S7 Fig. Correlation coefficient values (Spearman or Pearson) were depicted as superscript letters “a” or “b”, respectively. P values represent the statistical significance of linear regression slopes in each group. Bioenergetic capacity (OXPHOS) values represent the respiratory rates data induced by ADP and calculated by subtracting the ADP rates by their equivalent "leak" values shown in S2 Table (for isolated mitochondria) and S3 Table (for flight muscle). Statistical analyses on bioenergetic capacity in isolated mitochondria were performed by using Mann Whitney test (indicated by superscript asterisks) as well as ANOVA and *a posteriori* Tukey´s test (indicated by superscript symbols) for flight muscle. Significant differences in isolated mitochondria were * *p<0*.*001* relative to G3P and PC+Mal, and ** *p<0*.*005* relative to PC+Mal. In flight muscle, significant difference was * *p<0*.*001* relative to G3P.
(PDF)
