# Supplementary figures and images for: Mitochondrial Physiology in the Major Arbovirus Vector Aedes aegypti: Substrate Preferences and Sexual Differences Define Respiratory Capacity and Superoxide Production
Source: PLoS One. 2015 Mar 24;10(3):e0120600. doi: 10.1371/journal.pone.0120600 (PMC4372595; doi:10.1371/journal.pone.0120600)

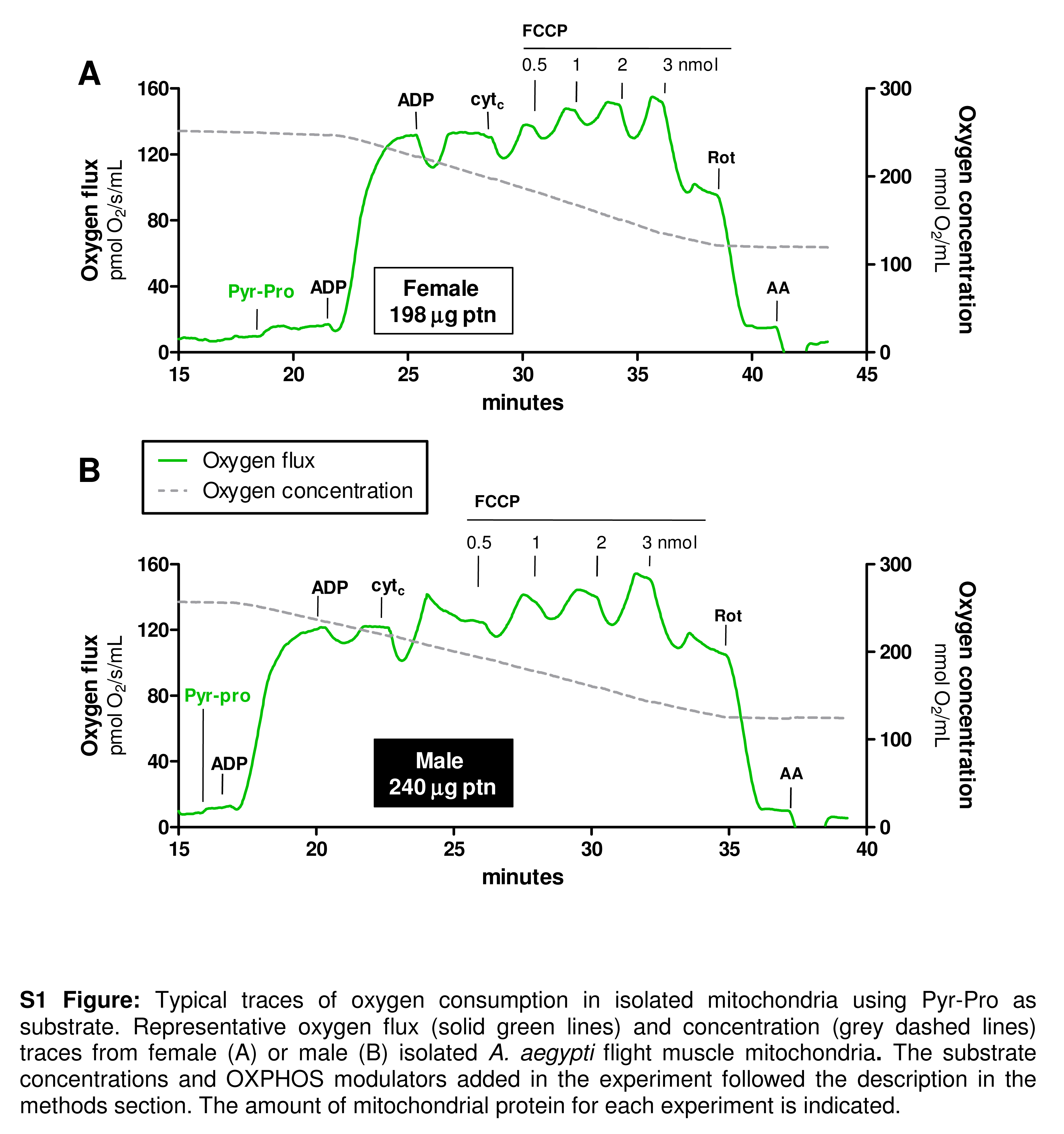

Supplement: S1 Fig — Representative oxygen flux (solid green lines) and concentration (grey dashed lines) traces from female (A) or male (B) isolated A. aegypti flight muscle mitochondria. The substrate concentrations and OXPHOS modulators added in the experiment followed the description in the methods section. The amount of mitochondrial protein for each experiment is indicated. (TIF) [file pone.0120600.s001.tif]

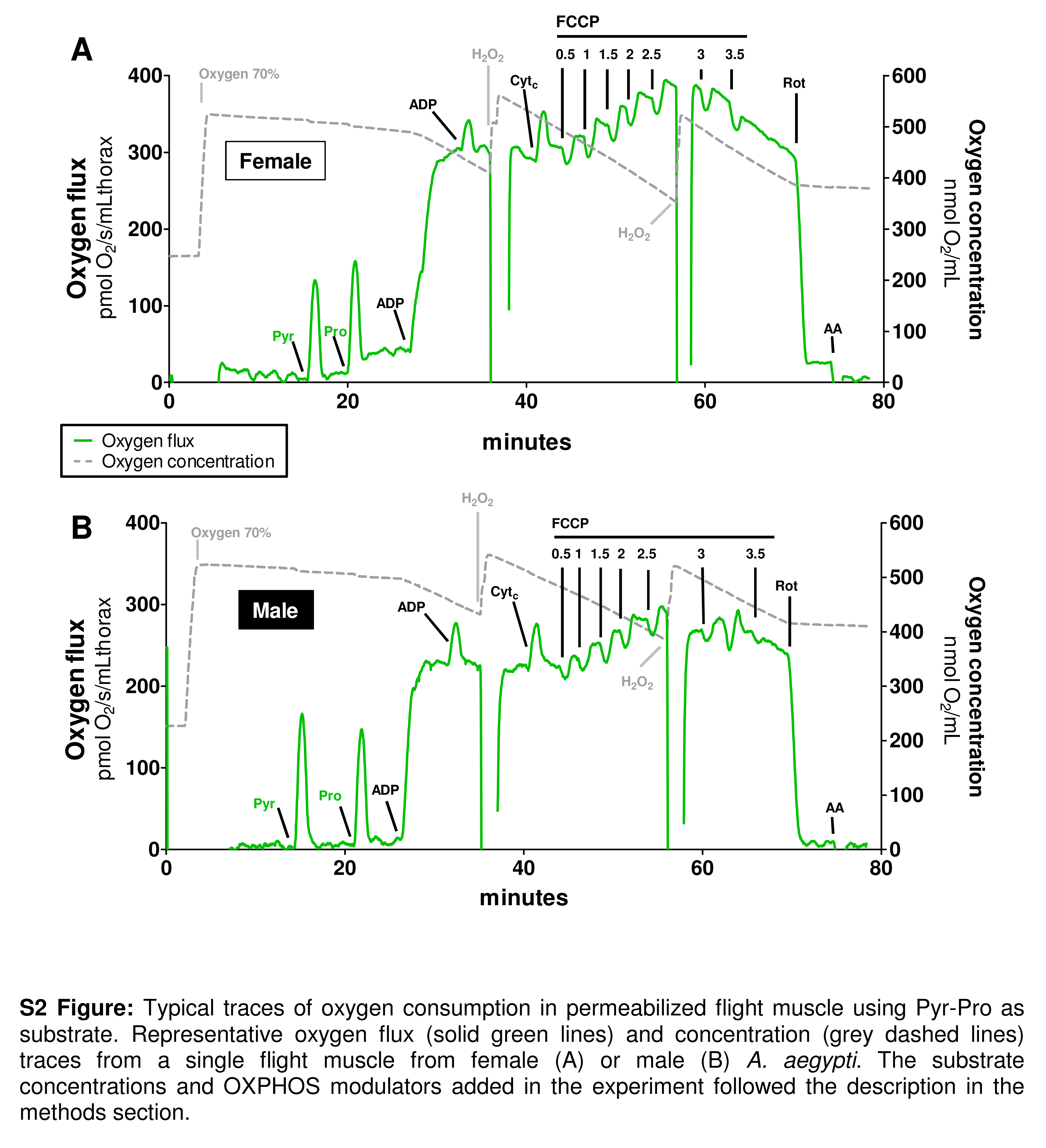

Supplement: S2 Fig — Representative oxygen flux (solid green lines) and concentration (grey dashed lines) traces from a single flight muscle from female (A) or male (B) A. aegypti. The substrate concentrations and OXPHOS modulators added in the experiment followed the description in the methods section. (TIF) [file pone.0120600.s002.tif]

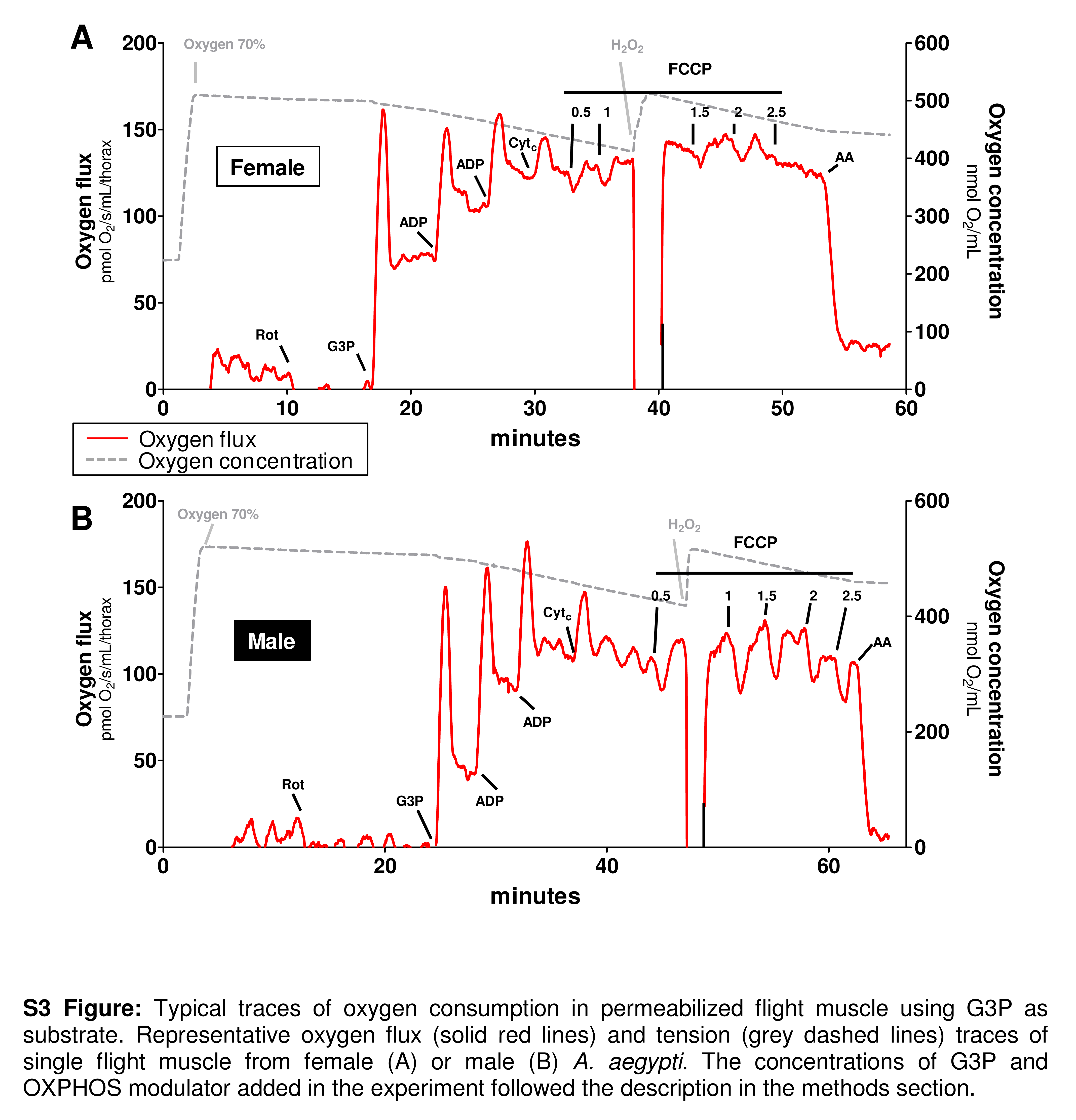

Supplement: S3 Fig — Representative oxygen flux (solid red lines) and tension (grey dashed lines) traces of single flight muscle from female (A) or male (B) A. aegypti. The concentrations of G3P and OXPHOS modulator added in the experiment followed the description in the methods section. (TIF) [file pone.0120600.s003.tif]

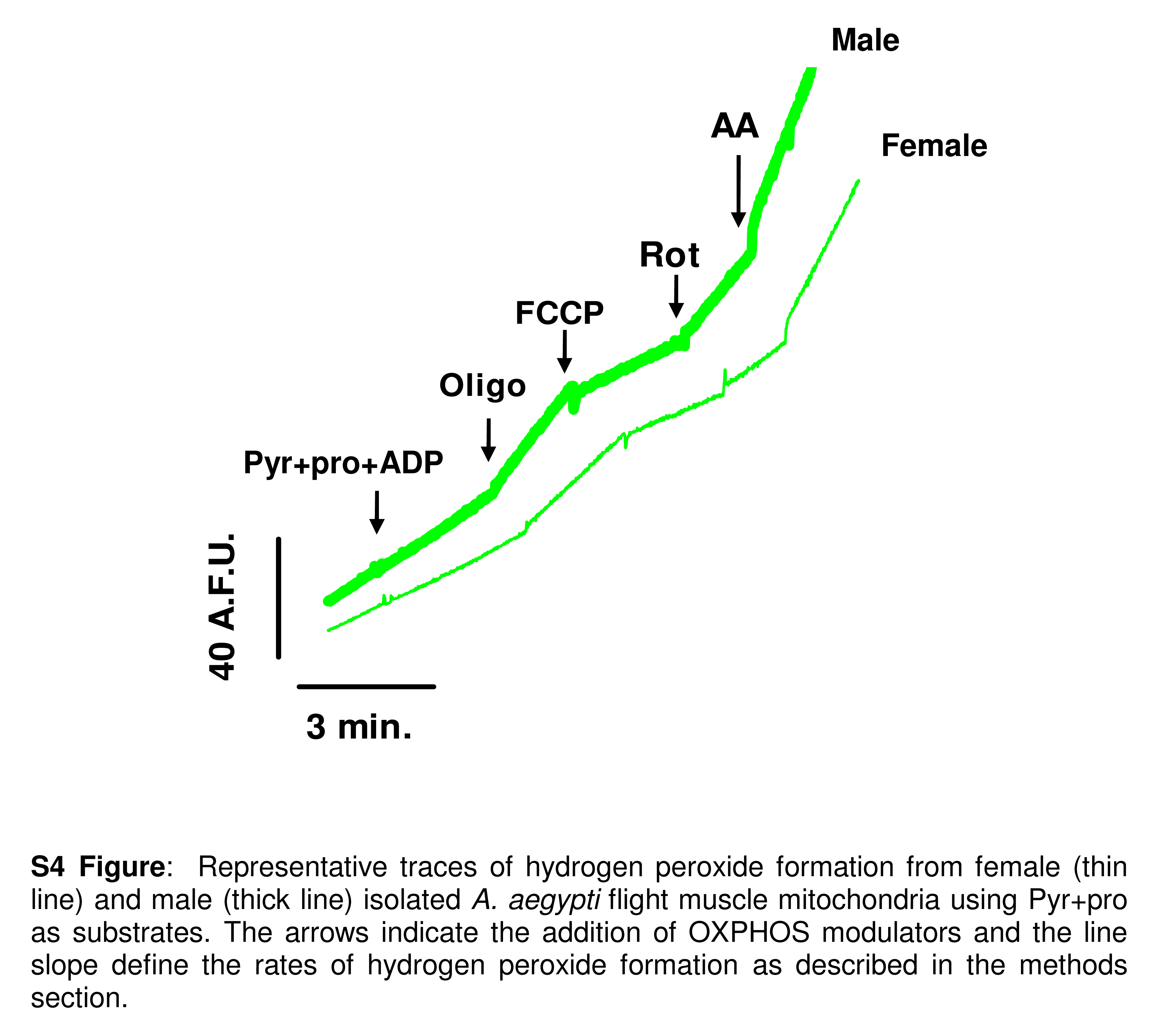

Supplement: S4 Fig — The arrows indicate the addition of OXPHOS modulators and the line slope define the rates of hydrogen peroxide formation as described in the methods section. (TIF) [file pone.0120600.s004.tif]

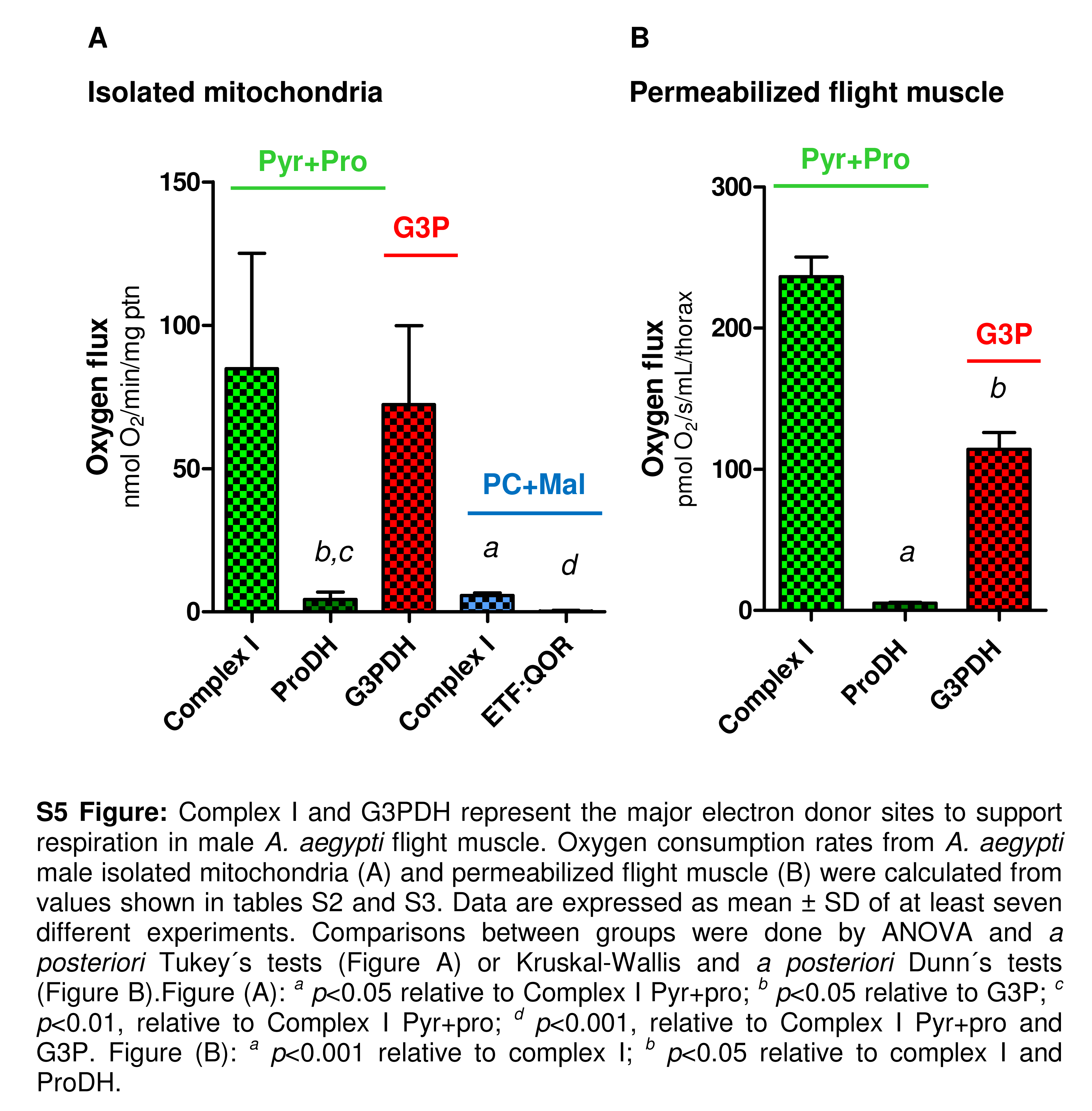

Supplement: S5 Fig — Oxygen consumption rates from A. aegypti male isolated mitochondria (A) and permeabilized flight muscle (B) were calculated from values shown in S2 and S3 Tables. Data are expressed as mean ± SD of at least seven different experiments. Comparisons between groups were done by ANOVA and a posteriori Tukey´s tests (Figure A) or Kruskal-Wallis and a posteriori Dunn´s tests (Figure B). Figure (A): a p<0.05 relative to Complex I Pyr+pro; b p<0.05 relative to G3P; c p<0.01, relative to Complex I Pyr+pro; d p<0.001, relative to Complex I Pyr+pro and G3P. Figure (B): a p<0.001 relative to complex I; b p<0.05 relative to complex I and ProDH. (TIF) [file pone.0120600.s005.tif]

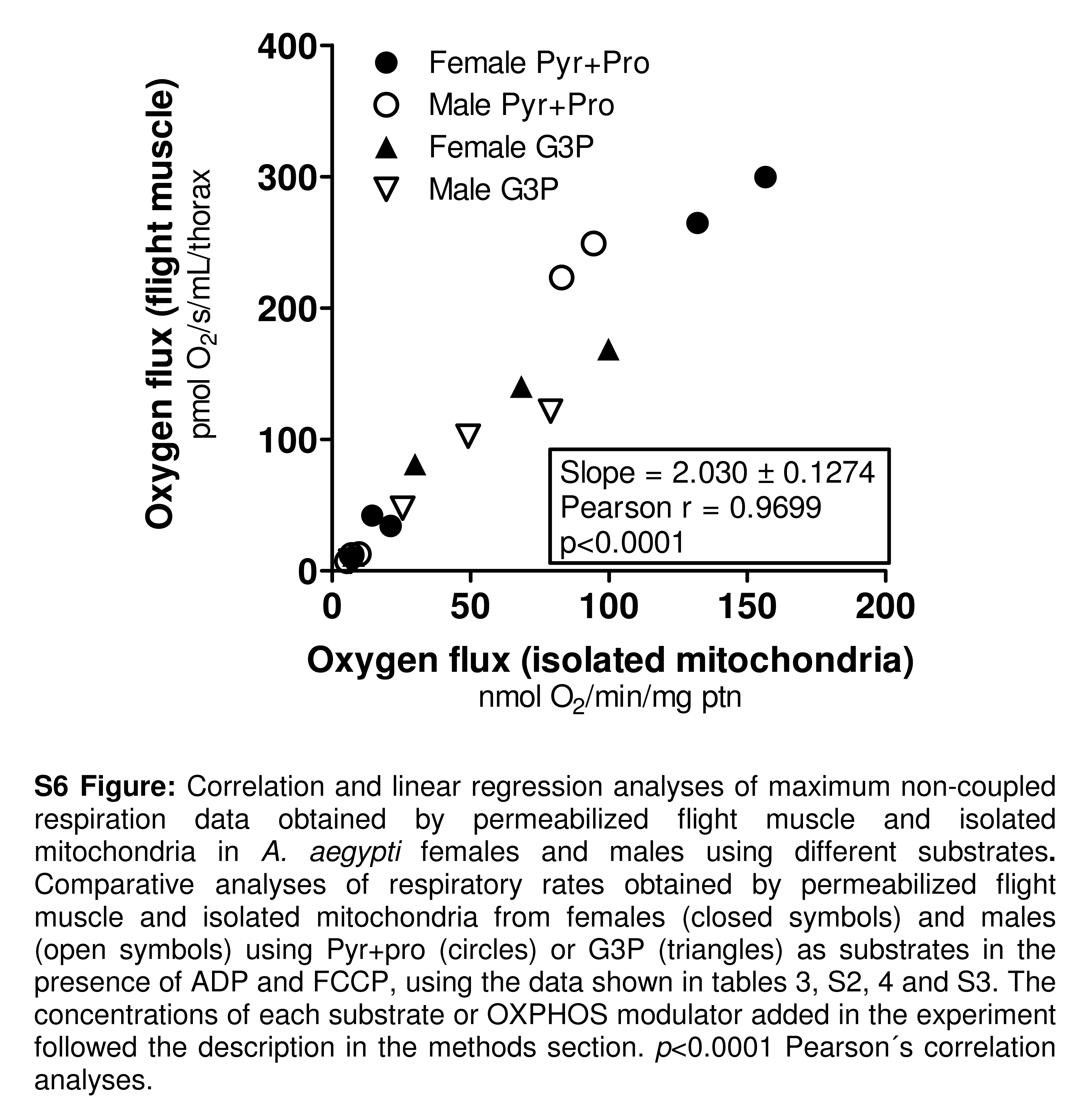

Supplement: S6 Fig — Comparative analyses of respiratory rates obtained by permeabilized flight muscle and isolated mitochondria from females (closed symbols) and males (open symbols) using Pyr+pro (circles) or G3P (triangles) as substrates in the presence of ADP and FCCP, using the data shown in Tables 3, S2, 4 and S3. The concentrations of each substrate or OXPHOS modulator added in the experiment followed the description in the methods section. p<0.0001 Pearson´s correlation analyses. (TIF) [file pone.0120600.s006.tif]

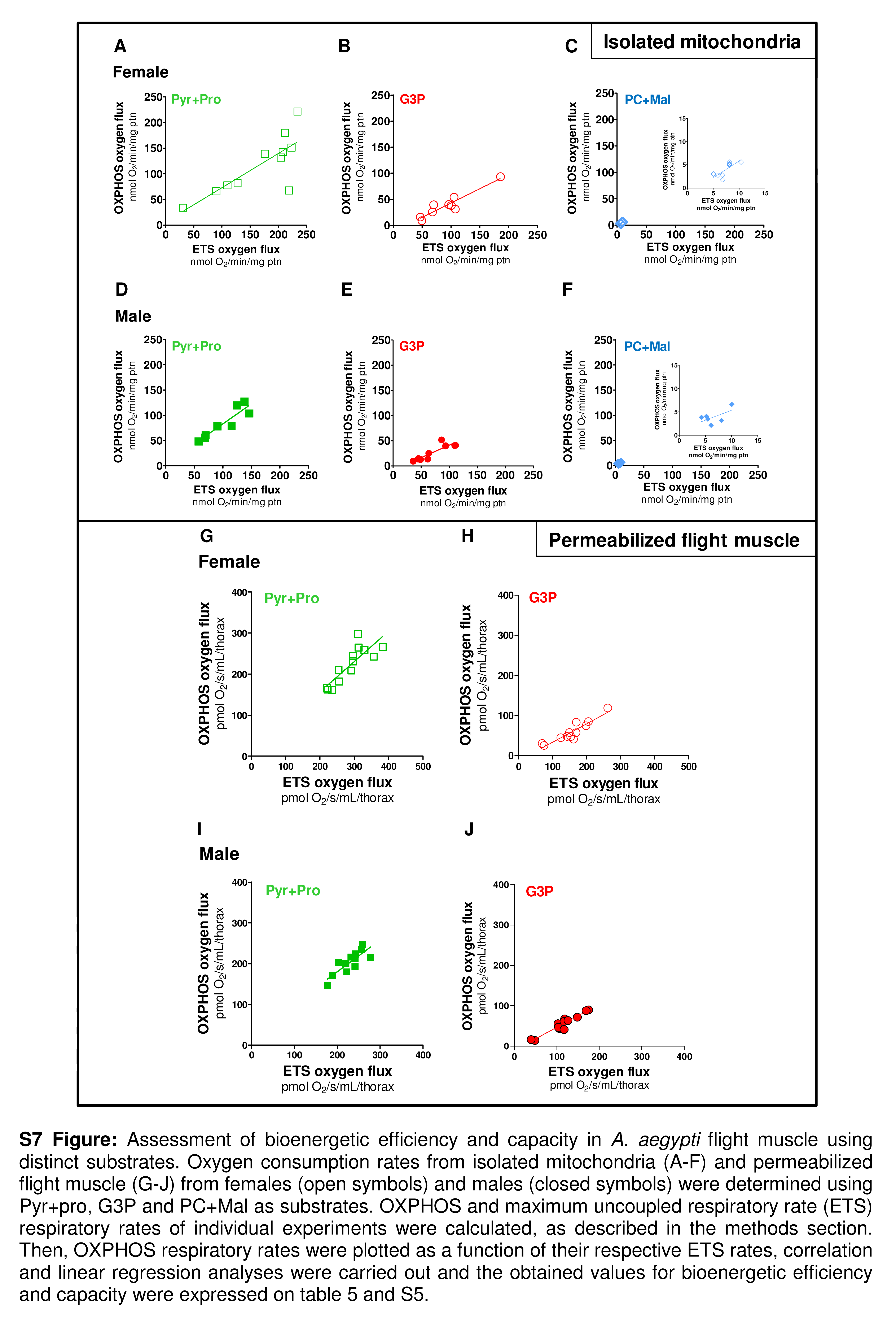

Supplement: S7 Fig — Oxygen consumption rates from isolated mitochondria (A-F) and permeabilized flight muscle (G-J) from females (open symbols) and males (closed symbols) were determined using Pyr+pro, G3P and PC+Mal as substrates. OXPHOS and maximum uncoupled respiratory rate (ETS) respiratory rates of individual experiments were calculated, as described in the methods section. Then, OXPHOS respiratory rates were plotted as a function of their respective ETS rates, correlation and linear regression analyses were carried out and the obtained values for bioenergetic efficiency and capacity were expressed on Table 5 and S4. (TIF) [file pone.0120600.s007.tif]

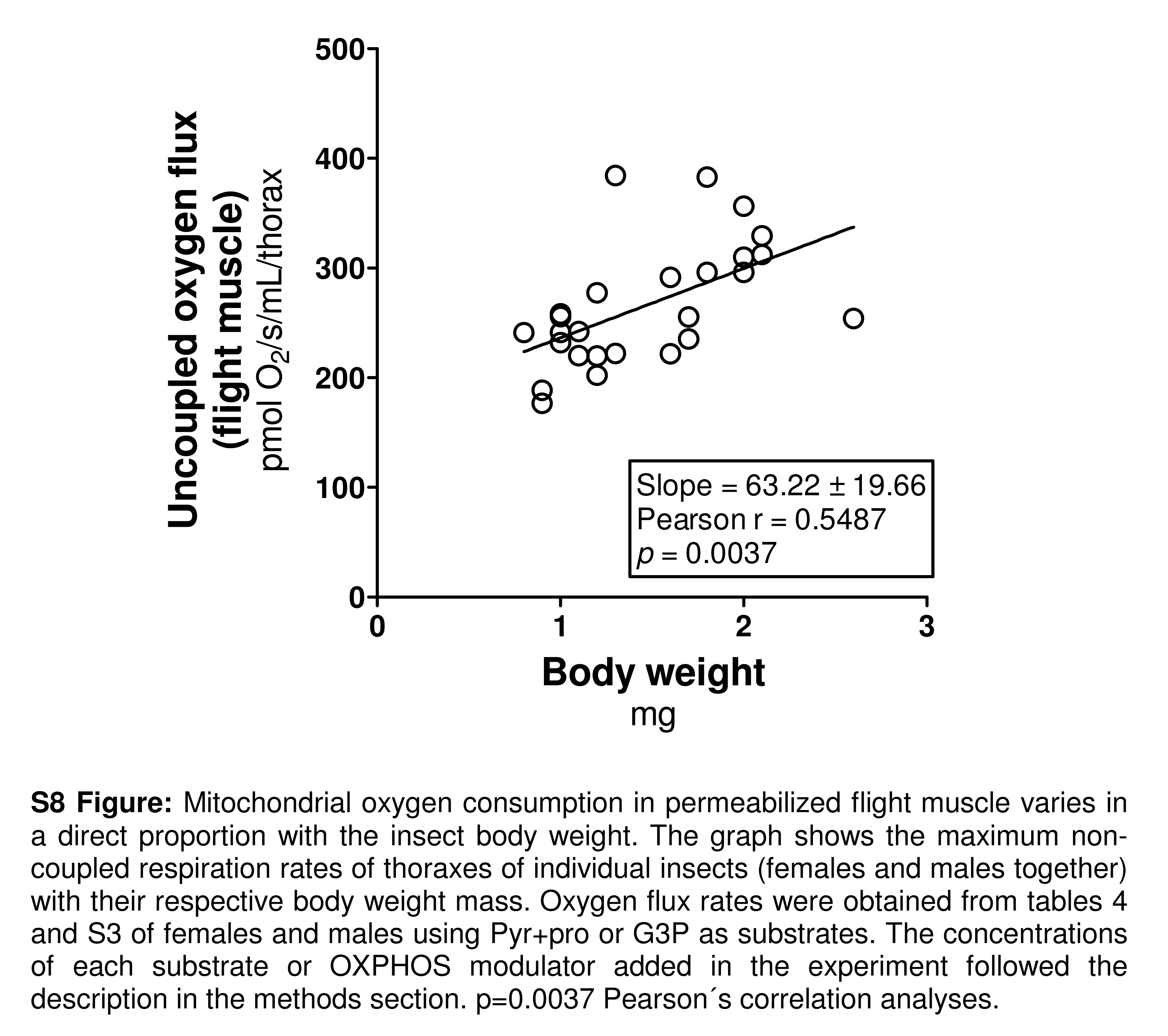

Supplement: S8 Fig — The graph shows the maximum non-coupled respiration rates of thoraxes of individual insects (females and males together) with their respective body weight mass. Oxygen flux rates were obtained from Tables 4 and S3 of females and males using Pyr+pro or G3P as substrates. The concentrations of each substrate or OXPHOS modulator added in the experiment followed the description in the methods section. p = 0.0037 Pearson´s correlation analyses. (TIF) [file pone.0120600.s008.tif]
