# Supplementary material for: Mitochondrial Physiology in the Major Arbovirus Vector Aedes aegypti: Substrate Preferences and Sexual Differences Define Respiratory Capacity and Superoxide Production
Source: PLoS One. 2015 Mar 24;10(3):e0120600. doi: 10.1371/journal.pone.0120600 (PMC4372595; doi:10.1371/journal.pone.0120600)
Supplement: S1 Table — Values were expressed as mean ± SD. Insect´s body weight was expressed as mg/insect. Data from wings length were expressed as mm and obtained from [2]. Data of wings area were expressed as mm2 and obtained from [55,56]. Mitochondrial protein yield was obtained from 120 insects (males or females) and was expressed as mg protein/mL of final preparations. Statistical analyses between groups were performed by using Mann-Whitney or unpaired t-test. a p<0.0001, b p<0.007 all relative to male. (PDF) [file pone.0120600.s009.pdf]

S1 Table: Sexual size dimorphism and flight muscle mitochondrial protein yield of *A. aegypti*		
	**Female**	**Male**
**Body weight**	1.88 ± 0.32 [a] (n=63)	1.06 ± 0.22 (n=62)
**Wings length**	3.85 ± 0.13 [a] (n=14)	2.59 ± 0.09 (n=13)
**Wings area**	1.98 ± 1.11 [b] (n=10)	1.03 ± 0.49 (n=10)
**Mitochondrial protein yield**	17.4 ± 5.72 (n=69)	16.0 ± 4.67 (n=70)

S1 Table: Sexual size dimorphism and flight muscle mitochondrial protein yield of *A. aegypti.* Values were expressed as mean ± SD. Insect´s body weight was expressed as mg/insect. Data from wings length were expressed as mm and obtained from [2]. Data of wings area were expressed as mm^2 and obtained from [55,56]. Mitochondrial protein yield was obtained from 120 insects (males or females) and was expressed as mg protein/mL of final preparations. Statistical analyses between groups were performed by using Mann-Whitney or unpaired t-test. [a] $p<0.0001$, [b] $p<0.007$ all relative to male.
