# Supplementary material for: Mitochondrial Physiology in the Major Arbovirus Vector Aedes aegypti: Substrate Preferences and Sexual Differences Define Respiratory Capacity and Superoxide Production
Source: PLoS One. 2015 Mar 24;10(3):e0120600. doi: 10.1371/journal.pone.0120600 (PMC4372595; doi:10.1371/journal.pone.0120600)
Supplement: S2 Table — Values were expressed as mean ± SD of nmol oxygen consumed/min/mg protein with the following substrates: 10 mM pyruvate + 10 mM proline (Pyr+Pro), 20 mM sn glycerol-3 phosphate (G3P) or 10 μM palmitoylcarnitine + 5 mM malate (PC+Mal). Addition of OXPHOS modulators were indicated as "+" in the first column as following: 2 mM ADP (+ADP), 10 μM cytochrome c (not shown), 2 μM FCCP (+FCCP), 0.5 μM rotenone (+Rotenone), and finally 2.5 μg/mL antimycin A (+ Antimycin). For all G3P measurements, experiments started after the addition of 0.5 μM rotenone. Statistical analyses were carried out only between the groups of different substrates and mitochondrial metabolic state and were performed by using either Kruskal-Wallis test followed by a posteriori Dunn´s test (indicated by superscript letters) or by ANOVA and a posteriori Tukey´s test (indicated by superscript symbols). Significant differences in “Leak” were ** p<0.001 relative to Pyr+Pro and PC+Mal. In “ADP”, significant differences were * p<0.05 relative to Pyr+Pro, # p<0.001 relative to PC+Mal. In “FCCP”, significant differences were # p<0.001 relative to PC+Mal. (PDF) [file pone.0120600.s010.pdf]

S2 Table: Contribution of different substrates to respiration of isolated mitochondria from flight muscle of *A. aegypti* males						
**Metabolic state**	**Pyr+Pro**	n	**G3P**	n	**PC+Mal**	n
Leak	7 ± 2	10	25 ± 13 **	11	2 ± 1	7
+ ADP	83 ± 33 #	10	53 ± 27 *,#	11	5 ± 2	7
+ FCCP	94 ± 41 #	10	79 ± 30 #	11	6 ± 3	7
+ Rotenone	10 ± 5	10	-	11	0.7 ± 0.6	7
+ Antimycin	5 ± 3	10	7 ± 4	11	0.6 ± 0.4	6

S2 Table: Contribution of different substrates to respiration of isolated mitochondria from flight muscle of *A. aegypti* males. Values were expressed as mean ± SD of nmol oxygen consumed/min/mg protein with the following substrates: 10 mM pyruvate + 10 mM proline (Pyr+Pro), 20 mM *sn* glycerol-3 phosphate (G3P) or 10 µM palmitoylcarnitine + 5 mM malate (PC+Mal). Addition of OXPHOS modulators were indicated as "+" in the first column as following: 2 mM ADP (+ADP), 10 µM cytochrome c (not shown), 2 µM FCCP (+FCCP), 0.5 µM rotenone (+Rotenone), and finally 2.5 µg/mL antimycin A (+ Antimycin). For all G3P measurements, experiments started after the addition of 0.5 µM rotenone. Statistical analyses were carried out only between the groups of different substrates and mitochondrial metabolic state and were performed by using either Kruskal-Wallis test followed by *a posteriori* Dunn´s test (indicated by superscript letters) or by ANOVA and *a posteriori* Tukey´s test (indicated by superscript symbols). Significant differences in “Leak” were ** $p<0.001$ relative to Pyr+Pro and PC+Mal. In “ADP”, significant differences were * $p<0.05$ relative to Pyr+Pro, # $p<0.001$ relative to PC+Mal. In “FCCP”, significant differences were # $p<0.001$ relative to PC+Mal.
