# Supplementary material for: Mitochondrial Physiology in the Major Arbovirus Vector Aedes aegypti: Substrate Preferences and Sexual Differences Define Respiratory Capacity and Superoxide Production
Source: PLoS One. 2015 Mar 24;10(3):e0120600. doi: 10.1371/journal.pone.0120600 (PMC4372595; doi:10.1371/journal.pone.0120600)
Supplement: S3 Table — Values were expressed as mean ± SD of pmol O2/s/mL/thorax in five different mitochondrial metabolic states using: 10 mM pyruvate + 10 mM proline, 20 mM sn glycerol-3 phosphate, followed by the addition of 2 mM ADP (ADP), 10 μM cytochrome c (not shown), 2.5 μM FCCP, 0.5 μM rotenone, 2.5 μg/mL antimycin A. Statistical analyses were performed using Mann-Whitney test. a p<0.001 relative to Pyr+Pro. (PDF) [file pone.0120600.s011.pdf]

**S3 Table: Contribution of different substrates to sustain respiration in permeabilized flight muscle from *A. aegypti* males**				
**Metabolic state**	**Pyr+Pro**	n	**G3P**	n
Leak	12 ± 3	13	48 ± 12 [a]	12
+ ADP	223 ± 40	13	102 ± 36 [a]	12
+ FCCP	249 ± 51	13	122 ± 42 [a]	12
+ Rotenone	13 ± 4	13	-	12
+ Antimycin	7 ± 3	13	7 ± 3	12

S3 Table: Contribution of different substrates to sustain respiration on permeabilized flight muscle of *A. aegypti* males. Values were expressed as mean ± SD of pmol O_2/s/mL/thorax in five different mitochondrial metabolic states using: 10 mM pyruvate + 10 mM proline, 20 mM *sn* glycerol-3 phosphate, followed by the addition of 2 mM ADP (ADP), 10 µM cytochrome c (not shown), 2.5 µM FCCP, 0.5 µM rotenone, 2.5 µg/mL antimycin A. Statistical analyses were performed using Mann-Whitney test. [a] $p<0.001$ relative to Pyr+Pro.
