# Supplementary material for: Mitochondrial Physiology in the Major Arbovirus Vector Aedes aegypti: Substrate Preferences and Sexual Differences Define Respiratory Capacity and Superoxide Production
Source: PLoS One. 2015 Mar 24;10(3):e0120600. doi: 10.1371/journal.pone.0120600 (PMC4372595; doi:10.1371/journal.pone.0120600)
Supplement: S4 Table — Values of bioenergetic efficiency (slope) were expressed as mean ± SD of OXPHOS versus maximum uncoupled respiratory rate linear regression and correlation analyses made in S7 Fig. Correlation coefficient values (Spearman or Pearson) were depicted as superscript letters “a” or “b”, respectively. P values represent the statistical significance of linear regression slopes in each group. Bioenergetic capacity (OXPHOS) values represent the respiratory rates data induced by ADP and calculated by subtracting the ADP rates by their equivalent "leak" values shown in S2 Table (for isolated mitochondria) and S3 Table (for flight muscle). Statistical analyses on bioenergetic capacity in isolated mitochondria were performed by using Mann Whitney test (indicated by superscript asterisks) as well as ANOVA and a posteriori Tukey´s test (indicated by superscript symbols) for flight muscle. Significant differences in isolated mitochondria were * p<0.001 relative to G3P and PC+Mal, and ** p<0.005 relative to PC+Mal. In flight muscle, significant difference was * p<0.001 relative to G3P. (PDF) [file pone.0120600.s012.pdf]

Table S4: Mitochondrial bioenergetic efficiency and capacity in *A. aegypti* males flight muscle using different substrates				
**Isolated mitochondria**				
	**Bioenergetic efficiency (Slope)**	**Correlation coefficient**	***p***	**Bioenergetic capacity (OXPHOS)**
**Pyr+Pro**	0.79 ± 0.14	0.91 [b]	0.0015	84 ± 30 *
**G3P**	0.50 ± 0.08	0.87 [b]	0.0002	27 ± 15 **
**PC+Mal**	0.42 ± 0.28	-0.03 [a]	0.21	4 ± 1
**Flight muscle**				
**Pyr+Pro**	0.69 ± 0.09	0.92 [a]	< 0.0001	203.3 ± 28.2 #
**G3P**	0.57 ± 0.05	0.96 [a]	< 0.0001	54.8 ± 24.1

S4 Table: Mitochondrial bioenergetic efficiency and capacity in *A. aegypti* males flight muscle using different substrates**.** Values of bioenergetic efficiency (slope) were expressed as mean ± SD of OXPHOS versus maximum uncoupled respiratory rate linear regression and correlation analyses made in figure S6. Correlation coefficient values (Spearman or Pearson) were depicted as superscript letters “a” or “b”, respectively. P values represent the statistical significance of linear regression slopes in each group. Bioenergetic capacity (OXPHOS) values represent the respiratory rates data induced by ADP and calculated by subtracting the ADP rates by their equivalent "leak" values shown in tables S2 (for isolated mitochondria) and S3 (for flight muscle). Statistical analyses on bioenergetic capacity in isolated mitochondria were performed by using Mann Whitney test (indicated by superscript asterisks) as well as ANOVA and *a posteriori* Tukey´s test (indicated by superscript symbols) for flight muscle. Significant differences in isolated mitochondria were * $p<0.001$ relative to G3P and PC+Mal, and ** $p<0.005$ relative to PC+Mal. In flight muscle, significant difference was * $p<0.001$ relative to G3P.
